# Cardiac specific PRMT1 ablation causes heart failure through CaMKII dysregulation

**DOI:** 10.1038/s41467-018-07606-y

**Published:** 2018-11-30

**Authors:** Jung-Hoon Pyun, Hyun-Ji Kim, Myong-Ho Jeong, Byeong-Yun Ahn, Tuan Anh Vuong, Dong I. Lee, Seri Choi, Seung-Hoi Koo, Hana Cho, Jong-Sun Kang

**Affiliations:** 1 Department of Molecular Cell Biology, Sungkyunkwan University School of Medicine, Suwon 16419, Korea; 2 Single Cell Network Research Center, Sungkyunkwan University School of Medicine, Suwon 16419, Korea; 3 Department of Physiology, Sungkyunkwan University School of Medicine, Suwon 16419, Korea; 4 Division of Cardiology, Department of Medicine, Johns Hopkins University School of Medicine, Baltimore, MD 21205 USA; 5 Division of Life Sciences, Korea University, Seoul 02841, Korea; 6 Samsung Biomedical Institute, Samsung medical center, Seoul 06351, Korea

## Abstract

Dysregulation of Ca^2+^/calmodulin-dependent protein kinase (CaMK)II is closely linked with myocardial hypertrophy and heart failure. However, the mechanisms that regulate CaMKII activity are incompletely understood. Here we show that protein arginine methyltransferase 1 (PRMT1) is essential for preventing cardiac CaMKII hyperactivation. Mice null for cardiac PRMT1 exhibit a rapid progression to dilated cardiomyopathy and heart failure within 2 months, accompanied by cardiomyocyte hypertrophy and fibrosis. Consistently, PRMT1 is downregulated in heart failure patients. PRMT1 depletion in isolated cardiomyocytes evokes hypertrophic responses with elevated remodeling gene expression, while PRMT1 overexpression protects against pathological responses to neurohormones. The level of active CaMKII is significantly elevated in PRMT1-deficient hearts or cardiomyocytes. PRMT1 interacts with and methylates CaMKII at arginine residues 9 and 275, leading to its inhibition. Accordingly, pharmacological inhibition of CaMKII restores contractile function in PRMT1-deficient mice. Thus, our data suggest that PRMT1 is a critical regulator of CaMKII to maintain cardiac function.

## Abstract (web summary)

The mechanisms that regulate the activity of Ca^2 +^/calmodulin-dependent protein kinase II (CaMKII) in the context of heart failure are incompletely understood. Here the authors show that protein arginine methyltransferase 1 (PRMT1) prevents cardiac hyperactivation of CaMKII and heart failure development by methylating CaMKII at arginine residues 9 and 275.

## Introduction

Multifunctional Ca^2+^/calmodulin-dependent kinase II (CaMKII) plays a nodal function in pathological cardiac stress associated with cardiac hypertrophy, dilated cardiomyopathy^1–3^, and heart failure^4,5^. Dysregulated CaMKII has been linked with cardiomyopathy by phosphorylating various proteins important for excitation–contraction coupling and cell survival including ion channels and Ca^2+^ homeostatic proteins, and transcription factors that elicit hypertrophic and inflammatory gene expression^6–10^. Thus, CaMKII can simultaneously affect mechanical and electrical properties of cardiac muscle cells^11^. Studies with transgenic mice for two major cardiac isoforms of CaMKII delta b and c have demonstrated their critical roles in cardiomyocyte hypertrophy and dilated cardiomyopathy^1,12^. Recently, it is reported that patients with end-stage idiopathic dilated cardiomyopathy and ischemic cardiomyopathy exhibit enhanced activity of CaMKII delta b and c^13^. Consistently, a growing number of studies reveal that CaMKII inhibition is beneficial for improving myocardial function and reducing arrhythmias^11,14–18^. Therefore understanding the regulatory mechanisms of CaMKII activity is critical for development of strategy to treat cardiac dysfunction and heart failure. CaMKII is primarily regulated through Ca^2+^/calmodulin (CaM) binding and it returns to an inactive conformation after Ca^2+^/CaM unbinding. However, post-translational modifications such as autophosphorylation of threonine 286/287 of CaMKII induced by Ca^2+^/CaM binding^19,20^ and the oxidation of paired methionines (281/282) of CaMKII in response to oxidative stress^21–23^ result in sustained, autonomous CaMKII activation independently of Ca^2+^/CaM and have been associated with pathological cardiac signaling^19^.

Protein methylation, along with phosphorylation, controls a variety of cellular functions through regulation of signaling pathways or gene expression^24–27^. Protein arginine methyltransferases (PRMTs) are enzymes that catalyze the transfer of a dimethyl group to arginine residues of target proteins^28,29^. In mammals, nine PRMTs have been characterized and PRMT1, originally identified as a histone H4 methyltransferase, methylates also several non-histone proteins and implicated in diverse cellular processes, including hepatic gluconeogenesis and skeletal muscle function^30–33^. The potential involvement of PRMT1 in cardiac function has been proposed by a study of the arginine to histidine mutation at residue 2834 of desmoplakin, which is associated with arrhythmogenic cardiomyopathy^34–36^. However, the in vivo evidence for a critical role of PRMT1 in cardiac function is currently lacking. In this study, we investigate the role of PRMT1 in cardiac function by using cardiac muscle-specific null mice for PRMT1. Cardiac PRMT1 null mice exhibit a sudden death with dilated cardiomyopathy, likely caused by CaMKII dysregulation.

In this study, we investigate the role of PRMT1 in cardiac function by using myocardium-specific null mice for PRMT1. Cardiac PRMT1 null mice exhibit dilated cardiomyopathy and contractile dysfunction within 2-months. PRMT1-depleted cardiomyocytes display hypertrophic responses, while PRMT1 overexpression protects against pathological responses triggered by phenylephrine. PRMT1 deficiency enhances levels of total and phosphorylated CaMKII, and CaMKII inhibition restores contractile function of PRMT1-deficient hearts. PRMT1 interacts with and methylates CaMKII at arginine 9 and 275 thereby inhibiting its activity. In conclusion, PRMT1 deficiency in cardiomyocytes causes CaMKII dysregulation resulting in dilated cardiomyopathy and heart failure.

## Results

### Cardiac-specific PRMT1 deletion causes dilated cardiomyopathy

**Fig. 1:**
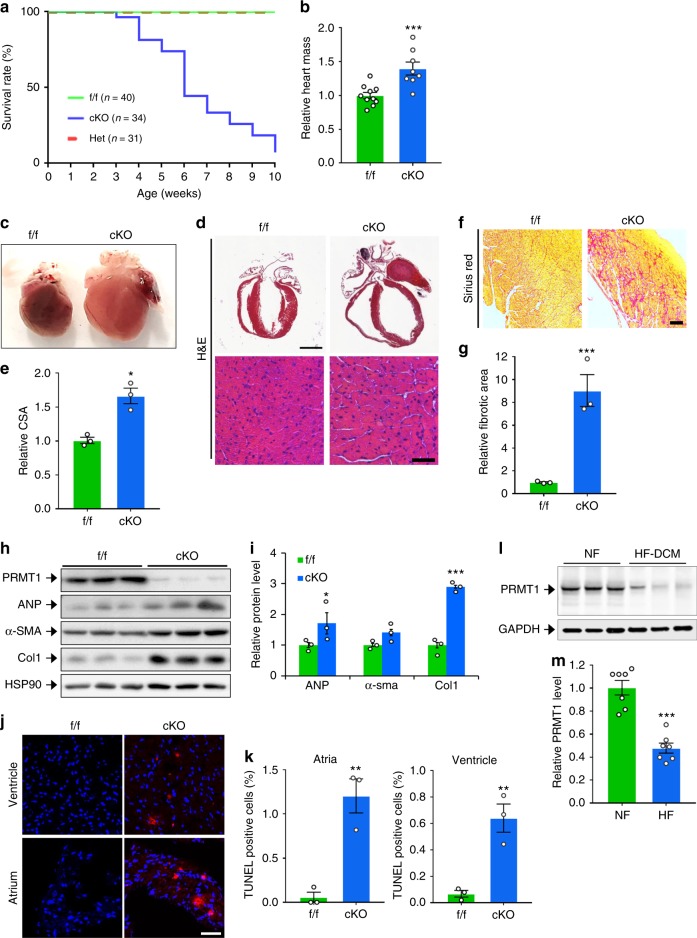
Cardiac-specific deletion of PRMT1 causes early lethality with dilated cardiomyopathy. **a** Survival rate of PRMT1^f/f^ (f/f, *n* = 40), PRMT1^f/cKO^ (Het, *n* = 31), PRMT1^cKO^ (cKO, *n* = 34) mice. **b** The relative heart mass to body weight of f/f (*n* = 8) and cKO (*n* = 8) mice. Data represent means ± SD. ^***^P *<* *0.001*, Student’s *t*-test. **c** A photograph of hearts from 8-weeks-old f/f and cKO mouse. **d** The histological analysis of hearts from part **c** by hematoxylin and eosin (H&E) staining. Lower panel: a high magnification of ventricular cardiomyocytes. Scale bar: 2 mm (upper panel), 100 μm (lower panel). **e** Quantification of cross sectional area (CSA) of ventricular cardiomyocytes shown in **d**. Data represent means ± SEM. ^*^P *<* *0.05*, Student’s *t*-test. *n* = 115–143 cells per group. **f** Sirius red staining of f/f and cKO. Scale bar: 100 μm. **g** The relative fibrotic area in whole heart sections as shown in **f**. Data represent means ± SEM. ^***^P *<* *0.001*, Student’s *t*-test. Mean values of all fields of hearts. *n* = 3. **h** Immunoblot analysis of 6–7-weeks-old f/f and cKO hearts for cardiac hypertrophic and fibrotic markers. α-SMA α-smooth muscle actin. **i** The relative signal intensity of proteins shown in **h**. Data represent means ± SD. ^*^P *<* 0.05, ^**^P *<* 0.01, ^***^P *<* 0.001, Student’s *t*-test. **j** The representative confocal microscopic image of TUNEL-positive nuclei (Red) in f/f and cKO hearts. Scale bar: 200 μm. **k** Quantification of apoptotic cells in atria and ventricle. Data represent means ± SD. ^**^P *<* 0.01, Student’s *t*-test. *n* = 8500–9305 cells from hearts per group. **l** Immunoblotting for PRMT1 in heart lysates from non-failing (NF) or heart failure (HF) human patients. **m** Quantification of PRMT1 protein levels in **l** (*n* = 7). Data represent means ± SD. ^*^P *<* 0.05, ^**^P *<* 0.01, ^***^P *<* 0.001, Student’s *t*-test

To investigate the role of PRMT1 in myocardium, PRMT1^fl/fl^ (designated f/f hereafter) mice are crossed with the myosin-heavy chain 6-Cre mice to generated PRMT1^fl/fl/Myh6-cre^ (designated cKO hereafter). In the control immunoblot, PRMT1 expression was specifically decreased in cardiac tissue. The residual PRMT1 expression likely is due to other cardiac cell types such as cardiac fibroblasts, as PRMT1 was detected in both cardiomyocytes (CM) and cardiac fibroblasts (CF) isolated from newborn rat hearts (Supplementary Fig. 1a, b). Male or female cKO mice were born normally without observable differences in body weights, relative to wild-type (WT) or heterozygous littermates (Supplementary Fig. 1c, d). However starting from postnatal 4-weeks of age, cKO mice exhibited lethality and almost all cKO mice die around 2-months of the postnatal life, while heterozygous mice did not show any abnormality at this age (Fig. 1a). These data suggest that the expression of PRMT1 in cardiomyocytes is critical for the survival of animals. The relative heart mass of 6–7-weeks-old cKO mice was significantly increased, without changes in body weight relative to f/f hearts (Fig. 1b). The relative lung weight to body weight was unchanged (Supplementary Fig. 1e). In addition, PRMT1 heterozygous mice exhibited roughly 25% lethality around 6 months of the age (Supplementary Fig. 1f).

A few days before death, cKO mice appeared sick and inactive, indicating a rapid progression toward overt heart failure. In the detailed analysis, 2-months-old cKO heart appeared severely dilated (Fig. 1c, d). The histological analysis revealed that the ventricular wall and interventricular septum are thinned and ventricular myocardial cells are enlarged (Fig. 1d, e). There were frequently larger spaces between cardiomyocytes in cKO hearts than in control hearts, indicating alterations in cell–cell contact (Fig. 1d). The sirius red staining disclosed a massive fibrosis in cKO myocardium (Fig. 1f, g). Furthermore, the protein level of fibrotic genes, atrial natriuretic peptide (ANP), and Collagen1 was substantially elevated in heart lysates from 6-weeks-old cKO mice, relative to that in f/f hearts (Fig. 1h, i). Unlike control cardiac tissue, ventricular and atrial tissue of cKO exhibited TUNEL-positive cells, suggesting for the underlying cell death contributing to dilated cardiomyopathy of cKO mice (Fig. 1j, k). Consistently, the level of PRMT1 protein was significantly reduced in ventricular myocardium from heart-failure patients, compared to non-failing hearts (Fig. 1l, m). Taken together, these data suggest that the myocardium-specific deletion of PRMT1 causes dilated cardiomyopathy with a severe fibrosis and cell death, eventually leading to heart failure.

### PRMT1-null cardiomyocytes exhibit cytoskeletal disruption

The immunostaining for cell interaction regulators and α-Actinin revealed profound changes in 8-weeks-old cKO cardiac tissue (Supplementary Fig. 2). The staining of junctional components, N-cadherin, desmoplakin and ZO-1 had become markedly more intense in cKO cardiomyocytes relative to that in control, suggesting for alterations in junctional integrity (Supplementary Fig. 2a–c). In addition, the wild-type cardiomyocytes showed well organized sarcomeric Z-line visualized by α-Actinin staining while cKO cardiomyocytes displayed severely perturbed Z-lines (Supplementary Fig. 2b). Immunostaining for the gap junction protein Cx43 also revealed mislocalization of Cx43 in the cytoplasm while it was decreased at the intercalated disc marked by N-cadherin, compared to f/f cardiomyocytes (Supplementary Fig. 2d, e). Transmission electron microscopy revealed the alterations in both cytoskeletal organization and mitochondrial morphology in cKO heart section (Supplementary Fig. 2f). These data suggest that PRMT1-deleted cardiomyocytes displayed cellular remodeling with alterations in cellular adhesion and cytoskeleton, and gap junction mislocalization.

### Cardiac-specific PRMT1 deletion impairs cardiac function

**Fig. 2:**
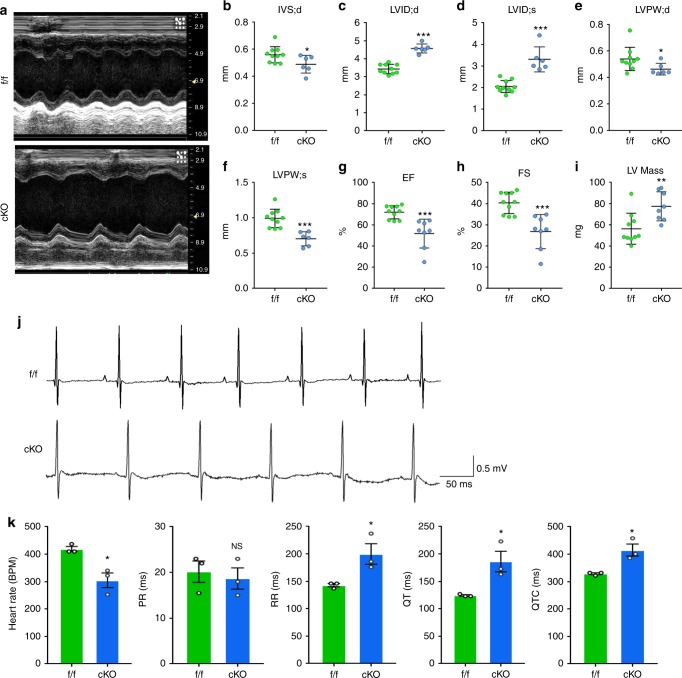
Cardiac-specific deletion of PRMT1 results in reduced cardiac function and increased mass. **a** The short axis transthoracic M mode echocardiographic tracings from 7-weeks-old f/f and cKO mice. **b**–**i** Echocardiographic parameters. IVS;d the interventricular septum;diastolic, LVID;d or LVID;s the internal dimension of left ventricle (LV); diastolic or systolic, LVPW;d or LVPW;sthe postwall thickness of LV; diastolic or systolic. EF the ejection fraction, FS the fraction shortening, LV Mass Left ventricular mass (*n* = 7–10 per group). Data represent means ± SEM. *P < 0.05, **P < 0.01, ***P < 0.001, Student’s *t*-test. **j** A representative ECG recording of spontaneous bradycardia in 7-weeks-old cKO and a normal ECG recording in f/f littermate. **k** Bar plots of heart rate and EKG intervals in f/f and cKO mice show the decreased heart rate and the prolonged QT interval in cKO mice. Data represent means ± SEM. *P < 0.05, Student’s *t*-test

To assess the contractile cardiac function, 6-weeks-old control and cKO mice were subjected to echocardiogram analysis to assess cardiac function. cKO mice had decreased interventricular septum thickness at end-diastole (IVS;d), left ventricular posterior wall thickness at end-diastole (LVPW;d), left ventricular posterior wall thickness at end-systole (LVPW;s), while both left ventricular internal dimension at end-diastole (LVID;d) and left ventricular internal dimension at end-systole (LVID;s) were significantly increased (Fig. 2a–i). Furthermore, cKO exhibited impaired contractility with decreased ejection fraction and fraction of shortening and increased LV mass, compared to the control. In addition, electrocardiography (ECG) showed that cKO mice exhibited sinus bradycardia and increased QT intervals (Fig. 2j–k), implying that cKO mice have important electrical changes commonly observed in cardiomyopathy. To examine the significance of the reduced PRMT1 levels in cardiac dysfunction triggered by cardiac stress, 2-months-old WT and heterozygous mice were subcutaneously injected with isoproterenol for 14 days, followed by the analysis of cardiac phenotype and function (Supplementary Fig. 3). The isoproterenol injection induced cardiac hypertrophy in heterozygous mice with reduced cardiac function, compared to the WT mice. These data suggest that PRMT1 deficiency causes cardiac dysfunction and cardiomyopathy.

### PRMT1 knockdown induces NRVM hypertrophy

**Fig. 3:**
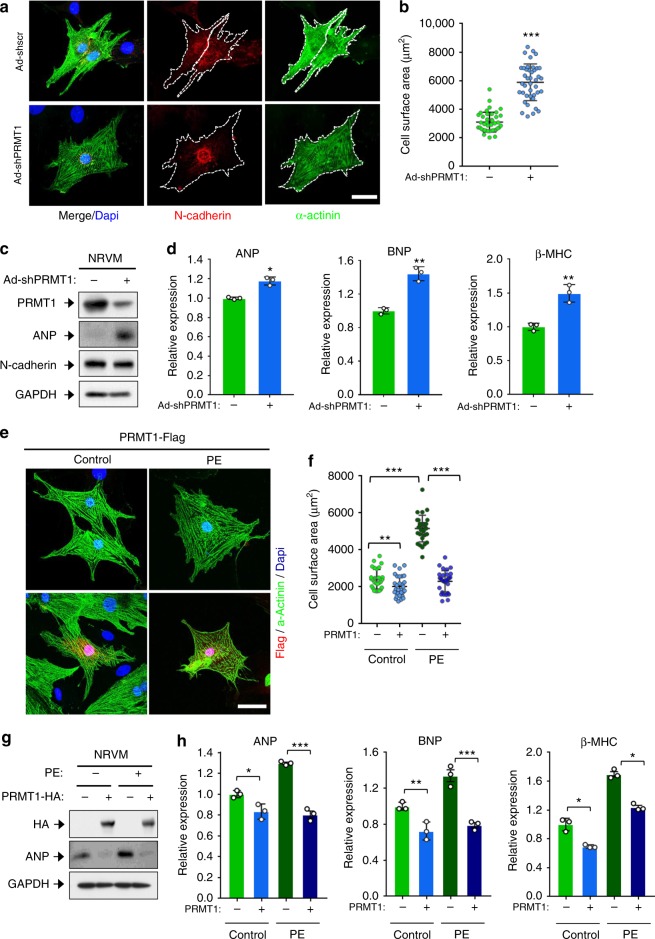
PRMT1 depletion causes cardiomyocyte hypertrophy while its overexpression prevents phenylephrine-induced hypertrophy. **a** Representative immunostaining images of NRVM cells infected with Ad-shscr (adenovirus-sh scrambled RNA) and Ad-shPRMT1 (adenovirus-PRMT1 shRNA). The white dashed line indicates a single cell surface. Scale bar: 50 μm. **b** Quantification of cell surface area as shown in **a**. *n* = 82 (Ad-shscr); *n* = 70 (Ad-shPRMT1). Data represent means ± SD. ^***^P *<* 0.005, Student’s *t*-test. **c** Immunoblot analysis of NRVM/Ad-shscr or NRVM/Ad-shPRMT1 cells. **d** qRT-PCR analysis of ANP, BNP, and β-MHC expression in NRVM/Ad-shscr and NRVM/Ad-shPRMT1. *n* = 3. Data represent means ± SD. ^*^P *<* 0.05, ^**^P *<* 0.01, Student’s *t*-test. **e** Immunostaining images of PRMT1-Flag transfected NRVM cells (red staining) treated with control or phenylephrine (PE) for 48 h. Scale bar: 50 μm. **f** Quantification of the surface area of cells similar shown in **e**. *n* = 56 (vehicle-pcDNA); *n* = 54 (vehicle-PRMT1-flag); *n* = 52 (PE-pcDNA); *n* = 48 (PE-PRMT1-flag). Data represent means ± SD. ^*^P *<* 0.05, ^**^P *<* 0.01, ^***^P *<* 0.001, Student’s *t*-test. **g** Protein analysis of control or PRMT1-HA transfected NRVM cells treated with vehicle or PE for 48 h for PRMT1 and ANP. **h** qRT-PCR analysis of ANP, BNP, and β-MHC levels in control or PRMT1-HA transfected NRVM cells treated with vehicle or PE. *n* = 3. Data represent means ± SD. ^*^P *<* 0.05, ^**^P *<* 0.01, ^***^P *<* 0.001, Student’s *t*-test

To further define the role of PRMT1 in cellular hypertrophy, rat newborn ventricular cardiomyocytes (NRVM) were infected with adenovirus expressing control scrambled or PRMT1-shRNA, followed by immunoblotting and immunostaining. Adenoviral PRMT1-shRNA infected NRVM greatly increased in size, relative to the control virus infected cells (Fig. 3a, b). Similarly to PRMT1-deficient hearts, PRMT1 depletion by two different PRMT1-shRNAs in cardiomyocytes dramatically elevated ANP protein level (Fig. 3c and Supplementary Fig. 4). In addition, the mRNA expression of hypertrophy-related genes, ANP, BNP, and β-MHC was enhanced in PRMT1-depleted NRVMs (Fig. 3d). Conversely, overexpression of PRMT1 in NRVMs (Fig 3e, f) as well as H9C2 cardiomyocytes (Supplementary Fig. 5) led to a resistance to hypertrophy in response to phenylephrine (PE) treatment, compared to control cells. Furthermore, PRMT1 overexpression also decreased ANP protein levels (Fig. 3g) as well as mRNA levels for ANP, BNP, and β-MHC under the control condition and also attenuated their increase in response to PE (Fig. 3h). These data suggest that PRMT1 depletion causes cardiomyocyte hypertrophy and PRMT1 overexpression attenuates cardiomyocyte hypertrophy induced by PE.

### PRMT1 regulates CaMKII through interaction and methylation

**Fig. 4:**
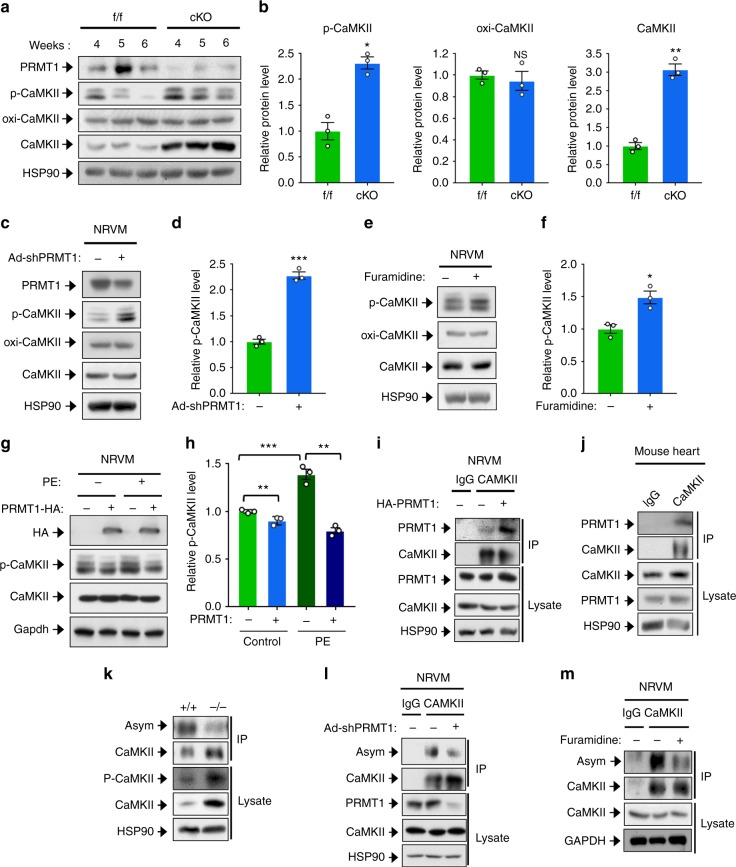
PRMT1 inhibits CaMKII through interaction and methylation. **a** Immunoblotting for p-CaMKII (phosphorylated-^Thr286/287^CaMKII), oxi-CaMKII (oxidized CaMKII), and total CaMKII in f/f and cKO mice between 4 to 6-weeks of age. **b** Relative p-CaMKII protein levels in 6-weeks-old hearts. *n* = 5. Data represent means ± SD. ^*^P *<* 0.05, ^**^P *<* 0.01, Student’s *t*-test. **c**, **d** Protein analysis of PRMT1-depleted NRVM and quantification of relative signal intensity. *n* = 3. Data represent means ± SD. ^***^P *<* 0.001, Student’s *t*-test. **e**, **f** Protein analysis of NRVM treated with a PRMT1-specific inhibitor furamidine for 4 h and quantification of relative signal intensity. *n* = 3. Data represent means ± SD. ^*^P *<* 0.05, Student’s *t*-test. **g** Analysis for CaMKII levels in control and PRMT1 overexpressing NRVM cells treated with PE for 1 day. **h** Quantification of relative protein levels similar experiments shown in **g**. *n* = 3. Data represent means ± SD. ^**^P *<* 0.01, ^***^P *<* 0.001, Student’s *t*-test. **i**, **j** Coimmunoprecipitation of PRMT1 with CaMKII in NRVMs and mouse heart. Immunoprecipitation with rabbit IgG serves as control. **k**, **l**, **m** Analysis of asymmetric dimethylation of CaMKII in wild-type and cKO heart lysates, PRMT1-depleted and furamidine-treated NRVM. Lysates were immunoprecipitated and immunoblotted with anti-Asym antibody

To investigate the molecular mechanism by which PRMT1 deficiency causes cardiac dysfunction and heart failure, f/f and cKO hearts from 4–6-weeks-old mice were subjected to immunoblot analysis for CaMKII activation, which is a key regulator for cardiac function. We confirmed no significant change in PRMT1 levels in this period (Supplementary Fig. 6). Total and the active, phosphorylated threonine 286/287-specific CaMKII (p-CaMKII) levels were elevated in cKO hearts, relative to control hearts (Fig. 4a). The quantification of CaMKII protein levels in 6-weeks-old cKO hearts revealed that the phosphorylated form and total CaMKII proteins increased roughly 2 and 3 folds, respectively, compared to control hearts (Fig. 4b). However, the Ca^2+^-calmodulin-independent oxidized CaMKII (oxi-CaMKII) levels did not alter. The quantitative reverse transcription PCR (RT-PCR) analysis for CaMKIIδ expression in cKO hearts and PRMT1-depleted NRVMs revealed that PRMT1 deficiency does not alter the transcript levels of CaMKII (Supplementary Fig. 7). Thus, the increase in CaMKII protein levels in cKO hearts is likely due to the increased protein stability.

To determine whether the CaMKII dysregulation observed in PRMT1 cKO hearts was cell-autonomous, the transient effect of PRMT1 depletion in NRVMs was analyzed. Consistently, PRMT1-depleted cardiomyocytes also showed greatly elevated levels of p-CaMKII, without changes in oxi-CaMKII and total CaMKII levels (Fig. 4c, d and Supplementary Fig. 4). To confirm whether PRMT1 can regulate CaMKII activity, the effect of a short-term, a 4 h-treatment with a PRMT1-specific inhibitor furamidine, a type I PRMT specific inhibitor MS023, or a pan PRMT inhibitor Adox was assessed in NRVM (Fig. 4e, f and Supplementary Fig. 8a–d). The inhibition of PRMT1 enhanced p-CaMKII levels without any change in total or oxi-CaMKII levels. In addition, control or HA-tagged PRMT1 overexpressing NRVMs were treated with vehicle or PE for 24 h and assessed for p-CaMKII levels (Fig. 4g, h). The level of p-CaMKII was increased in control-transfected cells in response to PE, while it was declined in PRMT1 overexpressing cells under both conditions. Taken together, these data suggest that PRMT1 might inhibit CaMKII activity in cardiomyocytes.

To assess whether methylation of CaMKII regulates CaMKII activity, we first examined the physical interaction between PRMT1 and CaMKII. Coimmunoprecipitation assays of control or HA-PRMT1-expressing NRVM cells and 293T cells cotransfected with HA-PRMT1 and Myc-CaMKII revealed that PRMT1 and CaMKII interacted (Fig. 4i and Supplementary Fig. 9a, b). Consistently, endogenous PRMT1 proteins were coimmunoprecipitated specifically with CaMKII in heart, while it did not with control IgG antibody (Fig. 4j). Interestingly, the interaction between endogenous PRMT1 and CaMKII was elevated in NRVM in response to PE treatment, likely contributing to control of CaMKII activation (Supplementary Fig. 9c). To further define the interaction between PRMT1 and CaMKII, four deletion constructs for CaMKII (aa1–270, catalytic domain; aa271–315, regulatory domain; aa380–533, association domain; aa271–533, regulatory-association domain) were constructed, followed by immunoprecipitation assay with PRMT1. The construct encoding regulatory domain failed to be expressed. Thus, this was omitted in the final experiment. The catalytic domain and regulatory-association domain interacted with PRMT1, while the association domain alone failed to be coprecipitated with PRMT1 (Supplementary Fig. 9d). Taken together, PRMT1 interacts with the catalytic and regulatory region of CaMKII.

We then examined the arginine methylation status of CaMKII in f/f and cKO hearts. The f/f and cKO heart lysates from 6-weeks-old mice were subjected to CaMKII immunoprecipitation and subsequent immunoblotting with antibody recognizing asymmetric dimethyl-arginine (Asym). Although CaMKII levels were elevated in PRMT1-deficient heart lysates, the level of Asym-positive CaMKII level was reduced and the methylated CaMKII level was inversely correlated with p-CAMKII level (Fig. 4k). To further define the role of methylation in the regulation of CaMKII activity, PRMT1 was inhibited by Ad-shPRMT1 or 4 h-treatment of furamidine and lysates were subjected to immunoprecipitation with CaMKII antibody. The Asym-CaMKII level was greatly reduced in PRMT1 inhibited NRVM (Fig. 4l, m). Taken together, these data suggest that PRMT1 interacts with and methylates CaMKII, likely resulting in suppression of CaMKII activation.

### CaMKII inhibition prevents remodeling of PRMT1 KO myocytes

**Fig. 5:**
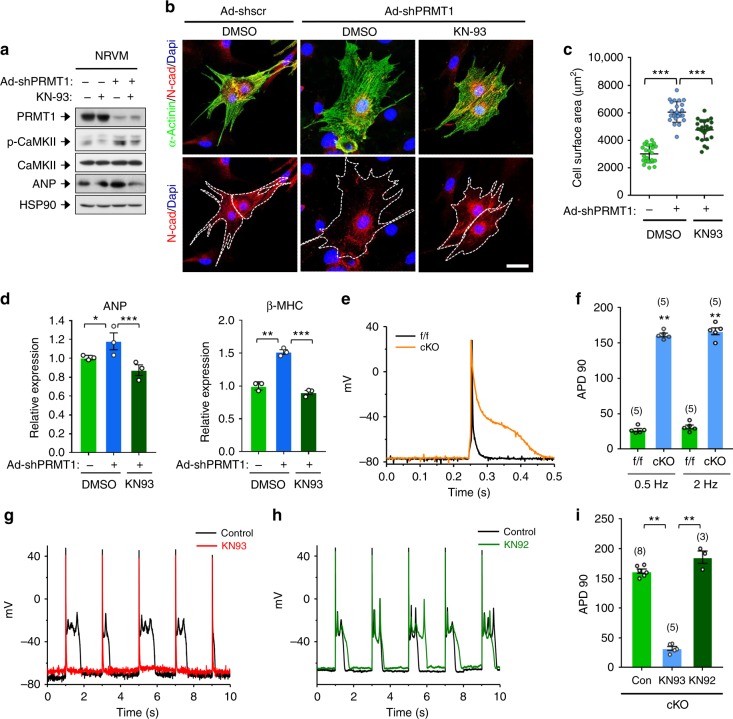
CaMKII inhibition by KN93 treatment prevents against pathological responses triggered by PRMT1 depletion. **a** CaMKII protein analysis of control or PRMT1-depleted NRVM cells treated with vehicle or KN93 for 24 h. **b** Representative images for NRVM cells immunostained with indicated antibodies. Scale bar: 50 μm. **c** Quantification of surface area of cells from similar experiments shown in **b**. *n* = 82 (Ad-shscr); *n* = 75 (Ad-shPRMT1); *n* = 78 (KN93-treated Ad-shPRMT1). Data represent means ± SD. ^***^P *<* 0.001, Student’s *t*-test. **d** qRT-PCR analysis of ANP, and β-MHC levels in control and PRMT1-depleted NRVM cells treated with vehicle or KN93. Data represent means ± SD. ^*^P *<* 0.05, ^**^P *<* 0.01, ^***^P *<* 0.001, Student’s *t*-test. **e** Representative AP traces in ventricular myocytes stimulated at 2 Hz from f/f and cKO mice. **f** Summary of the time required for 90% repolarization (APD90) showed that AP from cKO mice is dramatically prolonged. The APs were recorded from 7-weeks-old mice. *n* = 5. Data represent means ± SD. ^*^P *<* 0.05, ^**^P *<* 0.01, Student’s *t*-test. **g**–**h** Representative AP traces in ventricular myocytes stimulated at 0.5 Hz from cKO in the presence and absence of KN93 (**g**) or KN92 (**h**). **i** Bar graph showing the average APD90 of ventricular myocytes stimulated at 0.5 Hz from cKO mice in the absence and presence of KN93 or KN92. Data represent means ± SEM. **P *<* 0.01, Student’s *t*-test

Next, we determined the effect of CaMKII inhibition on cardiomyocyte hypertrophy caused by PRMT1 depletion. Control or PRMT1-depleted NRVMs were treated with DMSO or a CaMKII inhibitor KN93 for 24 h. We found that the KN93 treatment in PRMT1-depleted cells attenuated the increase in p-CaMKII level, similar to the control level (Fig. 5a). The enlargement in cell size caused by PRMT1 knockdown was significantly reduced by KN93 treatment (Fig. 5b, c). In addition, CaMKII inhibition restored the increase in ANP and β-MHC gene expression caused by PRMT1 knockdown (Fig. 5d). To validate the KN93 effect, control or PRMT1-depleted NRVM cells were treated with a CaMKII inhibitor peptide, AC3-I or control AC3-C for 12 h, followed by immunoblotting and qRT-PCR analysis (Supplementary Fig. 10). Similarly to the results with KN93 treatment, AC3-I treatment reduced ANP, BNP, β-MHC gene expression in PRMT1-depleted cells, while AC3-C had no significant effect, compared to PRMT1-depleted cells. These data suggest that CaMKII inhibition prevents cellular hypertrophy induced by PRMT1 depletion.

Because CaMKII induces abnormalities in cardiomyocyte depolarization and/or repolarization, we tested whether isolated myocytes from cKO hearts exhibited proarrhythmogenic after depolarizations on a cellular level. As depicted in action potential (AP) recordings and corresponding mean data in Fig. 5e–i, PRMT1 cKO resulted not only in increased AP duration but also in a higher incidence of early afterdepolarisations (EADs), which may arise from the > 5-fold increased AP duration. PRMT1 cKO did not change resting potential (–77.8 ± 1.8 (*n* = 5), –77.4 ± 2.1 (*n* = 5)mV for f/f, cKO mice, respectively; *P* > 0.05) or AP amplitude (105.8 ± 5.4 (*n* = 5), 104.8 ± 3.7 (*n* = 5) mV for f/f, cKO mice, respectively; *P* > 0.05). We then examined the ability of KN93 to suppress EADs in cKO cardiomyocytes. The examples shown in Fig. 5g indicate the ability of KN93 (1 μM) to suppress PRMT1 cKO-associated after depolarizations and APD prolongations. Similar results were seen in five cells. In contrast, the inactive analog of KN93, KN92 (1 μM) did not suppress cKO-related EADs and APD prolongation. These data show that cells from cKO mice having heart failure have an increased incidence of proarrhythmogenic EADs on a cellular level. Most importantly, CaMKII inhibition reduced those events pointing to the fact that CaMKII activity directly may contribute to electrical abnormalities in cKO mice.

### CaMKII inhibition improves cardiac function in PRMT1 cKO mice

**Fig. 6:**
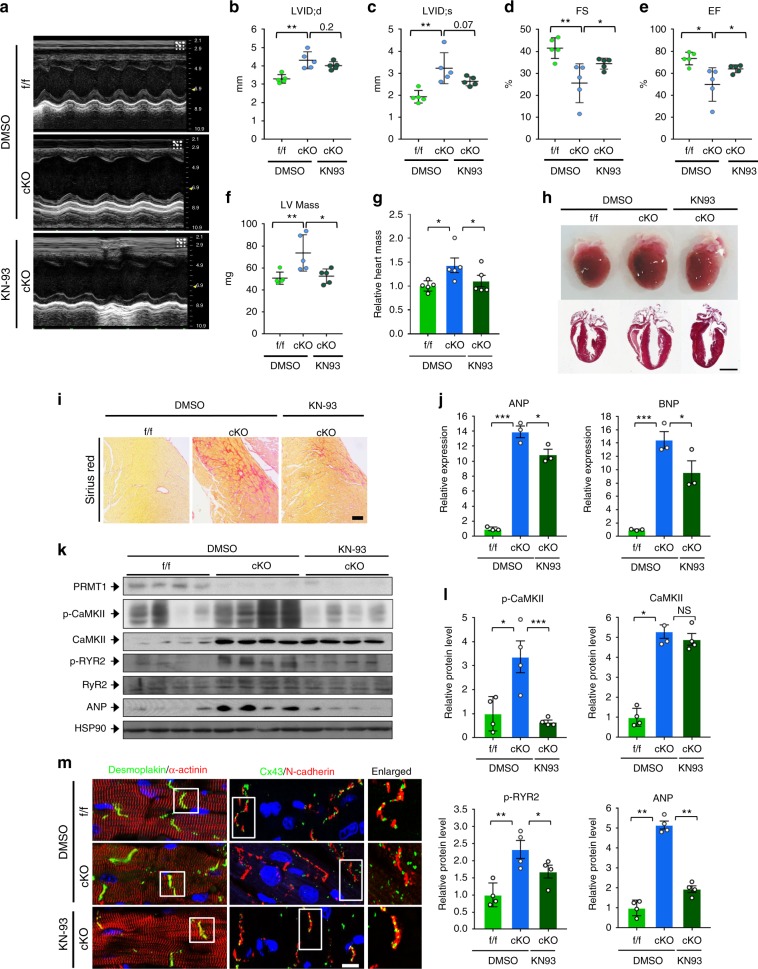
Inhibition of CAMKII protects PRMT1-deficient hearts from cardiomyopathy. **a** Representative echocardiogram of 5-weeks-old mice treated with vehicle or KN93 for 8 days. **b**–**f** Echocardiographic parameters of groups shown in **a**. LVID;d or LVID;s the internal dimension of left ventricle (LV); diastolic or systolic, EF the ejection fraction, FS the fraction shortening, LV mass Left ventricular mass. *n* = 5 per group. Data represent means ± SEM. ^*^P *<* 0.05, ^**^P *<* 0.01, ^***^P *<* 0.001, Student’s *t*-test. **g** Relative heart mass of 5-weeks-old f/f and cKO mice treated with vehicle or KN93 for 8 days. Data represent means ± SD. ^*^P *<* 0.05, ^**^P *<* 0.01, Student’s *t*-test. **h** Photograph of fleshly harvested hearts of f/f and cKO mice treated with vehicle or KN93 for 8 days (upper panel). H&E staining of whole heart sections of hearts shown in the upper panel. **i** Sirius red staining. Scale bar: 100 μm. **j** qRT-PCR analysis for hypertrophic genes, ANP and BNP. *n* = 5. Data represent means ± SD. ^*^P *<* 0.05, ^**^P *<* 0.01, ^***^P *<* 0.001, Student’s *t*-test. **k** Protein expression analysis of hearts from control DMSO-treated f/f and cKO, and KN93-treated cKO mice for 8 days. **l** Quantification of relative protein levels from **l**. *n* = 4. Data represent means ± SEM. ^*^P *<* 0.05, ^**^P *<* 0.01, ^***^P *<* 0.001, Student’s *t*-test. **m** Immunohistochemistry images of f/f and cKO cardiac sections. Scale bar: 50 μm

Based on these data, we hypothesized that cardiac dysfunction and dilated cardiomyopathy in PRMT1-deficient mice might be due to enhanced CaMKII activity. Since hearts from 4-weeks-old cKO mice showed CaMKII hyperactivation without obvious cardiac dysfunction, postnatal 4-weeks-old cKO mice were injected daily with vehicle DMSO or KN93 for total 8 days and then mice were subjected to echocardiogram (Fig. 6a–f). In line with the greater basal CaMKII activity in cKO mice, CaMKII inhibition improved cardiac function as shown in ejection fraction and fractional shortening. In addition, CaMKII inhibition reduced the left ventricular mass in cKO mice. Consistently, CaMKII inhibition decreased the relative heart mass to body weight in cKO mice almost to that of control f/f hearts (Fig. 6g), without changes in relative lung mass (Supplementary Fig. 11a). CaMKII inhibition also improved cardiac dilation of cKO mice (Fig. 6h). In addition, CaMKII inhibition also attenuated fibrosis in cKO hearts as assessed by sirius red staining (Fig. 6i and Supplementary Fig. 11b). Similarly to attenuated fibrosis, the increase of ANP and BNP levels was significantly blunted by CaMKII inhibition in cKO hearts (Fig. 6j, k). Next, we monitored the expression levels of key regulatory proteins involved in Ca^2+^ homeostasis (Fig. 6k, l). As expected, KN93 treatment reduced p-CaMKII levels without affecting total CaMKII levels in cKO hearts. Among CaMKII substrates, the phosphorylation level of ryanodine receptor 2 (RYR2)^37–39^ was increased in vehicle-treated cKO hearts compared to f/f hearts and this change was blunted by KN93 treatment. With KN93 treatment, ANP protein level of cKO hearts was also decreased (Fig. 6k, l). Furthermore, the structural assessment of cardiomyocytes showed an improvement in the myofibrillar arrangement and the localization of Cx43 at the intercalated discs (Fig. 6m). Taken together, these findings suggest that the inhibition of CaMKII activity restores cardiac function of cKO mice.

### PRMT1 inhibits CaMKII by methylating R9 and 275 of CaMKII

**Fig. 7:**
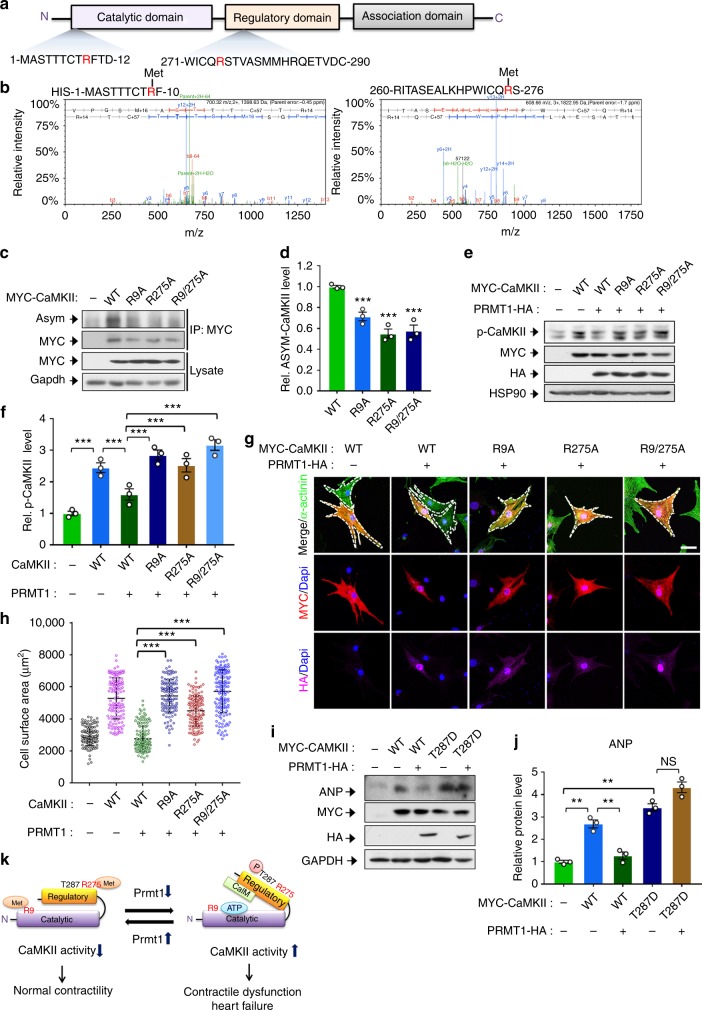
Arginine methylation of CaMKII at R9 and R275 is important for PRMT1-mediated suppression. **a** A schematic diagram of CaMKIId domain structure. **b** Identification of CaMKII methylation sites by mass spectrometry. His-tagged CaMKIId was coexpressed with PRMT1 in 293T cells and purified, followed by mass spectrometry analysis. The result indicated that arginine residues 9 and 275 of CaMKIId are methylated. **c** Asymmetric methylation levels of wild-type (WT) CaMKIId or arginine to alanine mutants at amino acid 9 or/and 275 (R9A, R275A, R9/275A). **d** Relative levels of asymmetric methylation of WT and mutants from experiments shown in **c**. *N* = 3. Data represent means ± SD. ^***^P *<* *0.001*, Student’s *t*-test. **e** Protein analysis of p-CaMKII levels of WT CaMKII or mutants transfected NRVM in the presence of PRMT1 overexpression. **f** Relative levels of p-CaMKII proteins from experiments shown in **e**. *n* = 3. Data represent means ± SD.^***^P *<* 0.001, one-way ANOVA. **g** Representative images for immunostaining of NRVM cells transfected with MYC-tagged WT CaMKII, mutant (Red) and HA-tagged PRMT1 (magenta). Cardiomyocytes were labeled with α-Actinin (green). White dash line indicates cell border. Size bar: 50 μm. **h** Quantification of the cell surface area from three independent experiments as shown in **g**. The cell number plotted in the graph per each lane is as following: *n* = 133 (control); 142 (WT + control); 140 (WT + PRMT1); 137 (R9A + PRMT1); 142 (R275A + PRMT1); 143 (R9/275A + PRMT1). Data represent means ± SD. ^***^P *<* 0.001, one-way ANOVA. **i** Immunoblotting for ANP expression in NRVM cells expressing WT or CaMKII T287D with PRMT1. **j** Quantification of ANP protein levels from experiments shown in **i**. *n* = 3. Data represent means ± SD. ^*^P *<* 0.05, Student’s *t*-test. **k** A working model summarizing the regulatory mechanism of CaMKII activity and cardiac function by PRMT1-mediated methylation of CaMKII at R9 and 275

To identify the methylation sites of CaMKII by PRMT1, HIS-tagged CaMKII delta and PRMT1 were coexpressed, purified and subjected to mass spectrometry analysis. The data indicated that arginine residues 9 in the catalytic domain and arginine residue 275 in the regulatory domain of CaMKII delta are methylated (Fig. 7a, b). To further assess, WT and arginine-to-alanine mutants of CaMKII delta (R9A, R275A, R9/275A) were expressed in NRVM and immunoprecipitated, followed by methylation analysis using Asym antibody (Fig. 7c, d). While WT proteins were readily detected by Asym antibody, mutants exhibited significantly reduced methylation. Consistently, the phosphorylated levels of WT CaMKII were decreased by PRMT1 overexpression, while PRMT1 overexpression failed to suppress mutants (Fig. 7e, f), suggesting that arginine methylation of CaMKII is important for PRMT1-mediated suppression. To examine the effect of mutants on cardiomyocyte hypertrophy, NRVM cells were transfected with control or PRMT1-HA in combination with MYC-WT or CaMKII mutant, followed by immunostaining for α-actinin, MYC and HA. The enlargement in cell size caused by WT CaMKII expression was blocked by PRMT1 coexpression, whereas cells expressing CaMKII RA mutants failed to respond to PRMT1 overexpression (Fig. 7g, h). To further examine the effect of CaMKII mutants on cardiomyocyte hypertrophy, NRVM cells were transfected with PRMT1-HA and CaMKII constructs and treated with PE for 12 h, followed by qRT-PCR for the level of ANP and BNP (Supplementary Fig. 12). Consistently, the coexpression of PRMT1 attenuated the increased expression of ANP and BNP induced by WT CaMKII in control and PE-treated NRVM. However, PRMT1 overexpression failed to reduce the expression of ANP and BNP induced by CaMKII mutants, suggesting that PRMT1 regulates CaMKII activity by arginine methylation. In a converse experiment, NRVM cells were transfected with WT CaMKII or an active form of CaMKII T287D mutant along with PRMT1. The expression of PRMT1 reduced ANP protein levels augmented by WT CaMKII, while the ANP level in cells expressing CaMKII T287D was not greatly altered by PRMT1 expression (Fig. 7i, j). Based on the data, we propose a working hypothesis that PRMT1 methylates CaMKII at arginine 9 and 275 thereby inhibiting its activation and controlling normal cardiac contractility (Fig. 7k). In summary, PRMT1 deficiency in cardiomyocytes causes dysregulation of CaMKII resulting in dilated cardiomyopathy and heart failure. Thus, PRMT1 is a key regulator of CaMKII activity in the control of cardiac function.

## Discussion

The main finding of this study is that PRMT1 is required for the suppression of CaMKII to ensure normal cardiac function. The ablation of PRMT1 function in myocardium results in a rapid progression to heart failure with dilated cardiomyopathy. PRMT1-deficient hearts exhibited various structural alterations such as cellular hypertrophy, fibrosis, elevated cytoskeletal regulators, and Cx43 mislocalization that resemble morphological and histological alterations characteristic of human cardiomyopathies^40–42^. The transient knockdown of PRMT1 in rat cardiomyocytes elicits the hypertrophic response as observed by hypertrophy-related gene expression and cellular enlargement while PRMT1 overexpression prevents hypertrophy in response to PE treatment, suggesting that PRMT1 is more directly involved in the suppression of hypertrophic response. This study provides compelling evidences that PRMT1 depletion induces myocardial hypertrophy and heart failure via CaMKII dysregulation. First, cKO hearts show drastically increased levels of total and phosphorylated CaMKII proteins. Second, PRMT1directly interacts with and methylates CaMKII in the heart. Third, CaMKII inhibition prevents hypertrophic responses to PRMT1 depletion in isolated cardiomyocytes. Finally, CaMKII inhibition protects against severe myocardial dysfunction in cKO mice. Injection of cKO mice with KN93 for 8 days markedly reversed the failing phenotypes, resulting in less left ventricular dilation and improved fractional shortening and ejection fraction, compared to severe failing phenotypes of control cKO mice. The recovery from cardiac dysfunction induced by CaMKII inhibition was also manifested by the decreased ANP and BNP expression, and fibrosis level of cKO mice. Taken together, these findings suggest that an increase in CaMKII activity is a direct cause of contractile dysfunction in cKO hearts. The current study is in agreement with a number of studies that have well established deregulated CaMKII as a key molecular abnormality in heart failure^2,11,43^.

In addition to heart failure, cKO mice have electrical abnormalities including sinus node dysfunction, increased QT interval and prolonged APD. Although heart failure is a mechanical problem, many heart failure patients experience arrhythmias, an electrical problem in which membrane excitability is inadequately controlled^11^. Growing evidence suggest that CaMKII plays a key role in heart failure and arrhythmias^1,15^. Excessive or deregulated CaMKII activity leads to phosphorylation of ion channels and Ca^2+^ cycling proteins that contribute to arrhythmias. Also, excess CaMKII can induce SA node dysfunction via sinus node cell death^44^. The present study significantly extends these observations by showing that CaMKII inhibition reverses APD prolongation and reduces EAD incidence in PRMT1-depleted cardiomyocytes. Interestingly, we observed upregulation of total CaMKII levels in cKO hearts along with hyperactive CaMKII while PRMT1-depletion or overexpression in cardiomyocytes modulated CaMKII activity without altering total CaMKII levels. Given that CaMKII can act as a proarrhythmic, feed-forward, signal in heart^45^, it is possible that arrhythmia and reduced cardiac function might contribute to the increased total CaMKII level in cKO mice, which might be underlying the discrepancy of total CaMKII levels in PRMT1-depleted cardiomyocytes. However we cannot exclude the possibility that PRMT1 depletion or overexpression might not result in efficient changes of CaMKII arginine methylation to elevated total CaMKII levels.

Since alterations in activities of Ca^2+^ cycling proteins or ion channels can induce the sustained increase in Ca^2+^/CaM, leading to constitutively active CaMKII, PRMT1 depletion might induce CaMKII activation via upregulation of Ca^2+^. Indeed, previous studies show that arginine methylation regulates sodium channel activity^46^ and ryanodine receptor^47^. It is well known that increased late Na^+^ current and abnormal Ca^2+^ leak through RyRs are involved in heart failure via Ca^2+^ overload in cardiomyocytes^48^. However, we ruled out this possibility by showing that APD prolongation and arrhythmogenic AP after depolarizations in PRMT1 null cardiomyocytes are reversed by CaMKII inhibition. These data suggest that ion channels responsible for AP in PRMT1 null cells might be targets of CaMKII rather than upstream of CaMKII activation. In addition, our data show that CaMKII inhibition with KN93 prevented the increased phosphorylation of RyR2 in cKO hearts, indicating a primary role for CaMKII in PRMT1 depletion-induced phosphorylation of Ca^2+^ cycling proteins. Taken together, these data suggest that CaMKII activation precedes the alteration of ion channel and Ca^2+^ cycling proteins. We also rule out the possibility that PRMT1 depletion induce an increase in ROS, which in turn causes oxidation of CaMKII because oxidation of CaMKII is unchanged in PRMT1-deficient myocytes. Thus, it is most plausible that arginine methylation may induce a conformational change in CaMKII or may reciprocally and competitively affect phosphorylation of CaMKII. Notably, arginine methylation of various proteins such as FoxO1, ERK, or p16 can modulate phosphorylation of adjacent serine residue^49–51^.We also found that the genetic or pharmacological inhibition of PRMT1 increases T287 phosphorylation of CaMKII in cardiomyocytes, while its oxidation level is unchanged. Supporting these results, PRMT1 overexpression inhibits CaMKII phosphorylation in response to PE. Taken together, these data suggest that the mechanism involving autophosphorylation likely is the regulatory target of PRMT1. In vitro kinase assay results confirmed higher autophosphorylation of R275A mutant, compared to WT CaMKII. However, R9A mutant exhibited a similar kinase activity to WT CaMKII (Supplementary Fig. 13a, b). In addition, the binding affinity to PRMT1 was not greatly altered in both mutants, compared to WT CaMKII proteins. Currently, we are not sure how R9 methylation regulates CaMKII activity (Supplementary Fig. 13c). Considering the complexity of CaMKII regulation, the R9 methylation in catalytic domain could be involved in other regulatory mechanisms such as autoinhibition control. To elucidate the exact mechanism, further study will be required.

Our data show that the level of PRMT1 protein is reduced in human heart failure, supporting the notion that the level of cellular PRMT1 must be precisely maintained and controlled for cardiac function. Interestingly, heterozygous cKO mice exhibited a partial lethality at 6 months of age and a sensitivity to isoproterenol-induced cardiac defects, further supporting for the importance of reduced PRMT1 in cardiac diseases. In this study we demonstrate a novel regulatory mechanism whereby PRMT1 regulates CaMKII activity and the overall contractile properties of the heart. This highlights the importance of arginine methylation as a key mechanism in experimental and human heart failure. The beneficial effects of PRMT1 on CaMKII in cardiomyocytes indicate that arginine methylation of CaMKII may provide a novel therapeutic strategy for the treatment of heart failure.

## Methods

### Reagents

Antibodies against p-CaMKII (1:500, ab32678), p-RyR2(S2808) (1:500, ab59225), RyR2 (1:200. ab117840), α-SMA (1:1000, ab5694), and Desmoplakin (1:200, ab106342) were purchased from Abcam. Flag-M2 (1:1000, F1804) and α-Actinin (1:1000, A7737) were obtained from Sigma-Aldrich. Antibodies against Gapdh (1:1000, LFPA0018) and HA (1:1000, LFMA0048) were from AbFrontier. Collagen1 (1:1000, SC-8784) and HSP90 (1:1000, SC-7947) were obtained from Santa Cruz Biotechnology. PRMT1 (1:500, 07-404) and Oxi-CaMKII (1:500, 07-1387) were from Millipore. N-cadherin antibody (1:500, 610921) was from BD biosciences. ANP (1:500, PA5-29559) and Zonula Occludens-1 (1:200, 40-2200) were from Thermo Fisher Scientific. Antibodies against ASYM (1:200, 13522), Connexin43 (1:1000, 3512) and CaMKII (1:1000, 3362) were from Cell Signaling Technology. Anti-mouse (1:5000, 115-035-033), anti-rabbit (1:5000, 111-035-003) IgG peroxidase-conjugated secondary antibodies were purchased from Jackson Immunoresearch Laboratories and anti-goat (1:5000, 81-1620) IgG peroxidase-conjugated secondary antibodies was ordered from Thermo Fisher Scientifics.

### Animals

PRMT1-floxed allele was obtained from EUCOMM and to generate cardiac-specific PRMT1 null mice, PRMT1^f/f^ mice were crossed with mice carrying Myosin-heavy chain 6-Cre transgene (Jackson Laboratory; Tg(Myh6-cre)2182Mds/J). To identify mice genotype, PCR analysis was performed with genomic DNA isolated from toe as previously described. The primers used for genotyping are listed in Supplementary Table 1. To assess the phenotypes and molecular analysis, hearts from f/f and cKO littermates were used. For KN93 (Millipore, 422711) treatment, 4-weeks-old cKO mice were used for daily intraperitoneal injection with the vehicle dimethyl sulfoxide (DMSO, Sigma-Aldrich) or 10 μmol/kg of KN93 diluted in saline for 8 days and then subjected to echocardiography. The animal experiments in this study were approved by the Institutional Animal Care and Use Committee of Sungkyunkwan University School of Medicine (SUSM) and complied with the animal experiment guidelines of SUSUM Ethics Committee. SUSM is an Association for Assessment and Accreditation of Laboratory Animal Care International (AAALAC International) accredited facility and abide by the Institute of Laboratory Animal Resources (ILAR) guide. To induce pathological hypertrophic stress, Isoproterenol (Sigma-Aldrich) was administered subcutaneously as dose of 15 mg/kg/day for 14 days. The control group was treated with same volume of isotonic saline.

### Human myocardial tissue

The study was approved by Research Ethics Committee at the University of Pennsylvania (Philadelphia, PA) and Johns Hopkins University (Baltimore, MD), and complied with their regulations. Written informed consents were obtained from all participants. Failing human hearts were procured at the time of orthotopic heart transplantation at the Hospital of University of Pennsylvania. Non-failing hearts were obtained at the time organ donation from cadaveric donors. In all cases, hearts were arrested in situ using ice-cold solution, transported on wet ice, and flash frozen in liquid nitrogen within 4 h of explantation. All samples were full-thickness biopsies obtained from the free wall of the left ventricles.

### Histology and Immunohistochemistry

For the histological analysis, fleshly dissected mouse hearts were fixed with 4% paraformaldehyde, embedded in paraffin, sectioned with 4 μm thickness, and stained with hematoxylin and eosin (H&E, BBC biochemical) and sirius red (Abcam, ab150681). For immunohistochemistry, paraffin embedded sections were rehydrated and processed through antigen retrieval with 20 μg/mL proteinase K (Roche, 03115887), followed by standard protocol for immunostaining. To assess cell death, heart sections were processed by using Click-iT TUNEL assay Alexa^594^ imaging assay kit (Invitrogen, C10246). Cell nucleus were counterstained with Dapi. The procedures of staining were performed according to the manufacturer’s protocols. For confocal microscopy, LSM-710 confocal microscope system (Carl Zeiss) was used.

### Isolation of adult mouse ventricular myocytes

To isolate ventricular myocytes, 2-weeks-old WT and cKO littermates were used. Ventricular myocytes were isolated by perfusion with a Ca^2+^-free normal Tyrode solution containing collagenase (Worthington, type 2, LS004177) on a Langendorff column at 37 °C as previously described^52^ with minor modifications. Isolated ventricular myocytes were kept in high K^+^, low Cl^−^ solution at 4 °C until use. Normal Tyrode solution contained (in millimoles per liter) 140 NaCl, 5 KCl, 1 MgCl_2_, 1.8 CaCl_2_, 10 Hepes, and 10 glucose, adjusted to pH 7.4 with NaOH. The Ca^2+^-free solution contained (in millimoles per liter) 140 NaCl, 5 KCl, 1 MgCl_2_, 10 Hepes, and 10 glucose, adjusted to pH 7.4 with NaOH. The high K^+^, low Cl^−^ solution contained (in millimoles per liter) only Ca^2+^-tolerant, rod-shaped myocytes with cross-striations and without spontaneous contractions or significant granulation were selected for electrophysiological experiments.

### Cell culture, transfection, infection, and Immunostaining

HEK293T (ATCC, CRL-3216) and H9C2 (KCLB, 21446) cells were cultured in 10% FBS (fetal bovine serum)  in DMEM (Dulbecco's Modified Eagle Medium) with antibiotics. NRVMs were isolated from postnatal day 1–2 Sparague-Dawley rat hearts and cultured as previously described^52^. Briefly, after washing and mincing the ventricular tissue in heart, tissue fragments were predigested by digestion buffer containing 0.1% collagenase Type 2 (Worthington), 0.1% trypsin and 1% glucose and gently agitated at 37 °C water bath. Isolated cell suspension was filtered through a cell strainer and centrifuged. The pelleted cells were resuspended and preplated into cell culture dish during 2 h for removing cardiac fibroblasts. Cell suspension was transferred to new culture dish and stabilized for 2 days in DMEM/F-12 containing 10% FBS, 5% horse serum and antibiotics. For transfection experiments, Lipofectamine2000 (Invitrogen, 11668) or Polyethylenimine (1 mg/mL, Sigma-Aldrich) were used.

To examine the effects of PRMT1 inhibition on CaMKII activity, cells were exposed to 200 μM of pan-PRMT inhibitor Adox (Sigma-Aldrich, A7154), 20 μM of PRMT1-specific inhibitor furamidine (Sigma-Aldrich, SML1559) or 1 μM of MS023 (ApexBio, B6183) for 4 h, followed by assessment of CaMKII phosphorylation. The expression vector for Myc-tagged CaMKII delta was kindly provided by Dr. Eric Olson (UT Southwestern). Arginine-to-alanine mutants of CaMKII delta for arginine residues 9 and/or 275 were generated by using quick-exchange mutagenesis kit (Stratagene, #200523). For examining whether suppression of CaMKII activity restore cardiomyocyte hypertrophy and electrical function, NRVM cells were treated with 10 μM CaMKII inhibitor KN93 (Millipore, 422711) or 50 μM of myristoylated CaMKII inhibitory peptide AC3-I (Autocamtide-3 derived inhibitory peptide, Myr–KKALHRQEAVDAL, ANASPEC, AS-64930). The control peptide AC3-C (Autocamtide-3 derived control peptide, Myr–KKALHAQERVDAL) was synthesized by Cosmogenetech.

Adenoviruses expressing control sh-scrambled (shscr) and shPRMT1(5'-GCAACTCCATGTTTCACAATC-3', targeting murine and rat PRMT1) were generated by homologous recombination between pAD-Easy vetor and linearized transfer pAD track vector as previously described^30^. To PRMT1 depletion experiments, NRVM or H9C2 cells were infected with adenovirus for 48 h and the infection efficiency was analyzed by GFP.

Immunostaining was carried out as previously described^53^. Briefly, cells were cultured on coverslip coated with gelatin. Fixation of cells was performed by the use of 4% PFA during 15 min. Cells were permeabilized with 0.1% Triton X-100 diluted in PBS, and then blocking with 2% BSA (Bovine serum albumin, cell nest, CNB 102-0250). Primary antibodies were diluted in blocking solution and incubated with cells overnight at 4 °C. Cells were washed with PBS and incubated with Fluor conjugated secondary antibodies 1 h at room temperature. Mowiol mounting solution was used on mounting.

### RNA extraction and quantitative PCR analysis

Total RNA was isolated from mouse hearts and NRVM using TRIzol (Invitrogen, 15596026) according to the manufacturer’s protocol. cDNA samples were synthesized with PrimeScript RT reagent kit (TaKaRa, RR064A) according to the manufacturer’s instruction and gene expression was analyzed with qPCR using SYBR premix Ex Taq (TaKaRa, RR420) on Thermal Cycler Dice Real Time System machine (TaKaRa, TP800) according to the manufacturer’s instruction. Primer sequences used in each reaction are listed in Supplementary Table 1.

### Protein analysis

Protein analysis was carried out as previously described^53,54^. Briefly, homogenized mouse hearts and cultured cells were lysed using RIPA buffer and analyzed by standard western blotting. The quantification of protein levels was obtained by the signal density analysis using image J (NIH) program and normalized to the loading control level. Immunoprecipitation was performed as previously described^55^. Briefly, tissues and cell cultures were lysed with extraction buffer containing Triton X-100 (protease inhibitor cocktail (Roche, 1183617001), pH 8.0; 150 mmol/L NaCl; 1 mmol/L EDTA; 1% Triton X-100; 10 mmol/L Tris-HCl). Precleared cell extracts were incubated with antibodies overnight at 4 °C and precipitated with Dynabeads (Invitrogen, 10003D) according to the manufacturer’s protocol. Clean-blot IP detection kit (Thermo scientific, 21232) was used for detecting target protein masked with IgG band resulted from immunoprecipitation. All uncropped scans of western blots are presented in Supplementary Figs. 14, 15, and 16.

For mass spectrometry, HIS-tagged CaMKII delta and PRMT1 were cotransfected into HEK293T cells and CaMKII proteins were purified by using Nickel-NTA resin (Novagen, 70666-4). Mass spectrometry analysis of purified HIS-CaMKII protein was performed by Medicinal Bioconvergence Research Center, Seoul National University. Mass spectrometry was carried out as described^56^.

### GST pull-down assay and in vitro kinase assay

GST pull-down assay was performed with using bacterially purified GST and GST-fused PRMT1 and MYC-tagged CaMKII proteins expressed in 293T cells. The GST fusion PRMT1 was immobilized on glutathione-sepharose beads by rotating for 1 h at 4 °C. The lysates containing MYC-tagged WT and mutant CaMKII proteins were added to spin column containing glutathione beads and gently rocking for 1 h at 4 °C. Columns were washed three times with lysis buffer and samples were eluted followed by western blotting analysis. For analyzing the autophosphorylation of WT and mutant CaMKII proteins, transiently transfected MYC-tagged WT, mutant CaMKII and HIS-tagged Calmodulin were purified by using MYC antibody and Ni-NTA beads, respectively. Purified CaMKII WT and mutant proteins were incubated in reaction buffer containing 0.5 μg of calmodulin, 150 mM ATP, and 1μCi gamma-^32^P ATP (Perkin Elmer Life Sciences) for 30 min at 30 °C. Samples were processed through SDS-polyacrylamide gel electrophoresis and autoradiography.

### Echocardiography and electrocardiography

For echocardiographic analysis, mice were anaesthetized with 1–2% (vol/vol) isoflurane. Echocardiography was performed on 4–8-weeks-old-mice and M-mode image derived from short axis of LV were recorded using visual sonics Vevo 2100 machine with 40-MHz probe (visual Sonics). Wall thickness, LV end-diastolic dimension (LVID;d), and end-systolic dimensions (LVID;s) were measured by M-mode tracing. The percentage of fractional shortening (FS) was calculated as equation: ([LVEDD-LVESD]/LVEDD) × 100, and ejection fraction (EF) percentage using the equation: ([EDV-ESV]/EDV) × 100, where EDV represents end-diastolic volume and ESV represents end-systolic volume. To record the electrocardiogram, mice were anesthetized with 2% (vol/vol) isoflurane, placed in the prone position on a warm surface essentially as previously published^52^. A Lead II ECG was recorded with s.c. electrodes using a PowerLab station (AD Instruments) and LabChart7 software. Heart rate and QRS interval duration were determined by averaging three consecutive beats during sinus rhythm.

### Electrophysiology

APs were recorded using a nystatin (200 μg/mL)-perforated patch-clamp configuration, with an EPC10 patch-clamp amplifier (Heka, Germany). Data were digitized and current injection (120–150pA, 9 ms) for AP generation were both controlled using Patch-Master software. The patch pipettes (World Precision Instruments) were made by a Narishige puller (Narishige, PP-830). The patch pipettes used had a resistance of 2–3MΩ when filled with the below pipette solutions. Normal Tyrode’s solution (143 mM NaCl, 5.4 mM KCl, 0.33 mM NaH_2_PO_4_, 5 mM HEPES, 0.5 mM MgCl_2_, 1.8 mM CaCl_2_, 11 mM D-glucose, pH adjusted to 7.4 with NaOH) was used as the bathing solution. The pipette solution for recoding APs contained 140 mM KCl, 10 mM HEPES, 5 mM EGTA, and 1 mM MgCl_2_, and the pH was adjusted to 7.2 with KOH. All recordings were carried out at 35 ± 1 °C.

### Statistical analysis

Data are represented as means ± SEM or ± SD as noted. The values were analyzed by the use of unpaired or paired two-tailed Student *t*-test. For multiple comparison, analysis of variance (ANOVA) test followed by Dunnett’s testing was used (GraphPad Prism software, version 7.00). Differences were considered statistically significant at *P* *<* 0.05.

## Electronic supplementary material

Supplementary Information

Reporting Summary

## Data Availability

All data supporting the findings in this study are available from the corresponding author upon reasonable request.
