# Supplementary material for: Cardiac specific PRMT1 ablation causes heart failure through CaMKII dysregulation
Source: Nat Commun. 2018 Nov 30;9:5107. doi: 10.1038/s41467-018-07606-y (PMC6269446; doi:10.1038/s41467-018-07606-y)
Supplement: Supplementary file 1 — Supplementary Information [file 41467_2018_7606_MOESM1_ESM.pdf]

Supplementary information

Cardiac specific PRMT1 ablation causes heart failure through CaMKII dysregulation

Jung-Hoon Pyun et al.

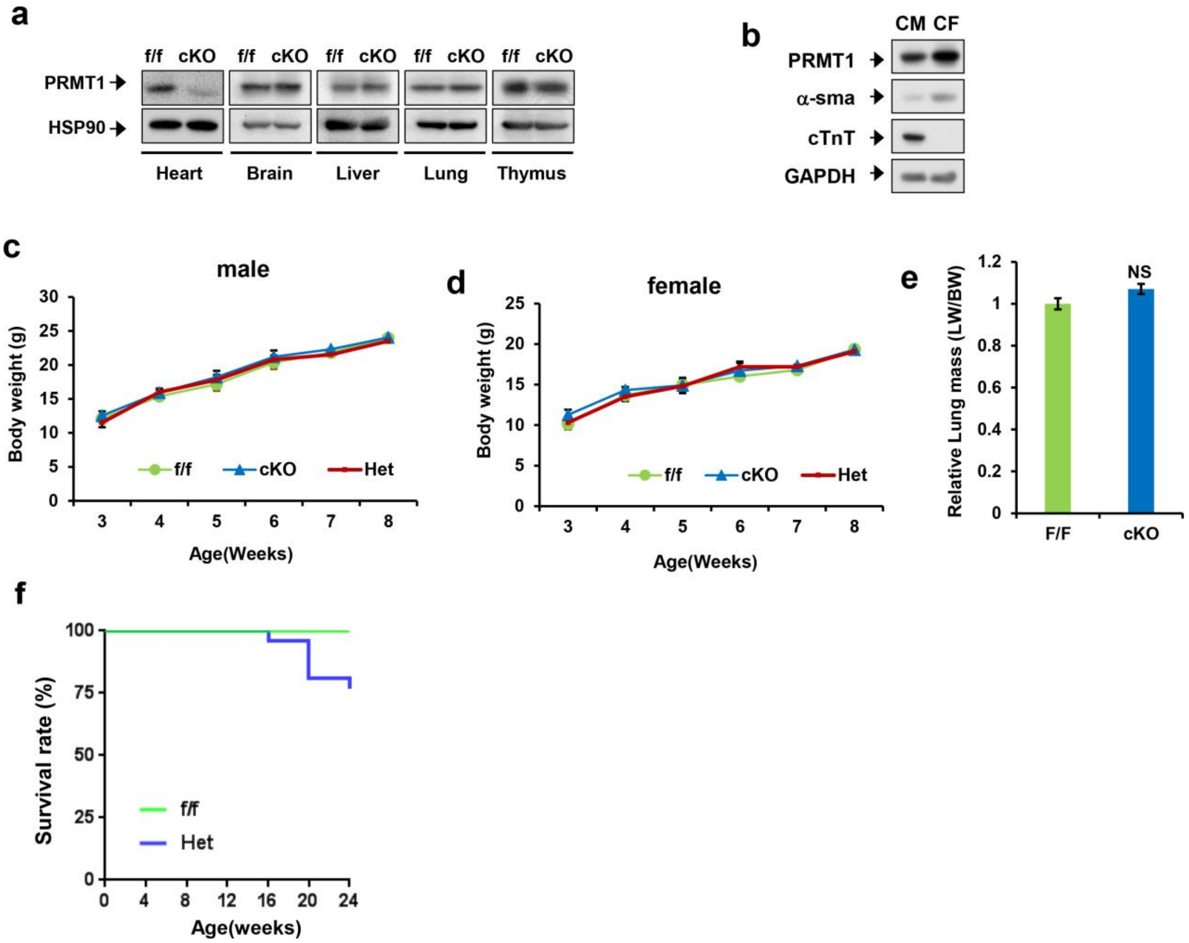

Supplementary figure 1.

(**a**) Protein analysis for PRMT1 expression in heart, brain, liver, lung and thymus isolated from f/f and cKO mice. (**b**) Immunoblotting for PRMT1 level in newborn rat cardiomyocytes (CM) and cardiac fibroblasts (CF). (**c, d**) Body weights of f/f , Het and cKO male and female mice from 3 weeks to 8 weeks. Data represent means ±SD. n=24. (**e**) Relative lung weight (LW)/body weight (BW) of 6-7 weeks-old f/f and cKO mice. n=5 for each group. Data represent means ±SD. (**f**) Survival rate of PRMT1$^{f/f}$ (f/f, n=30), PRMT1$^{f/cKO}$ (Het, n=30) mice.

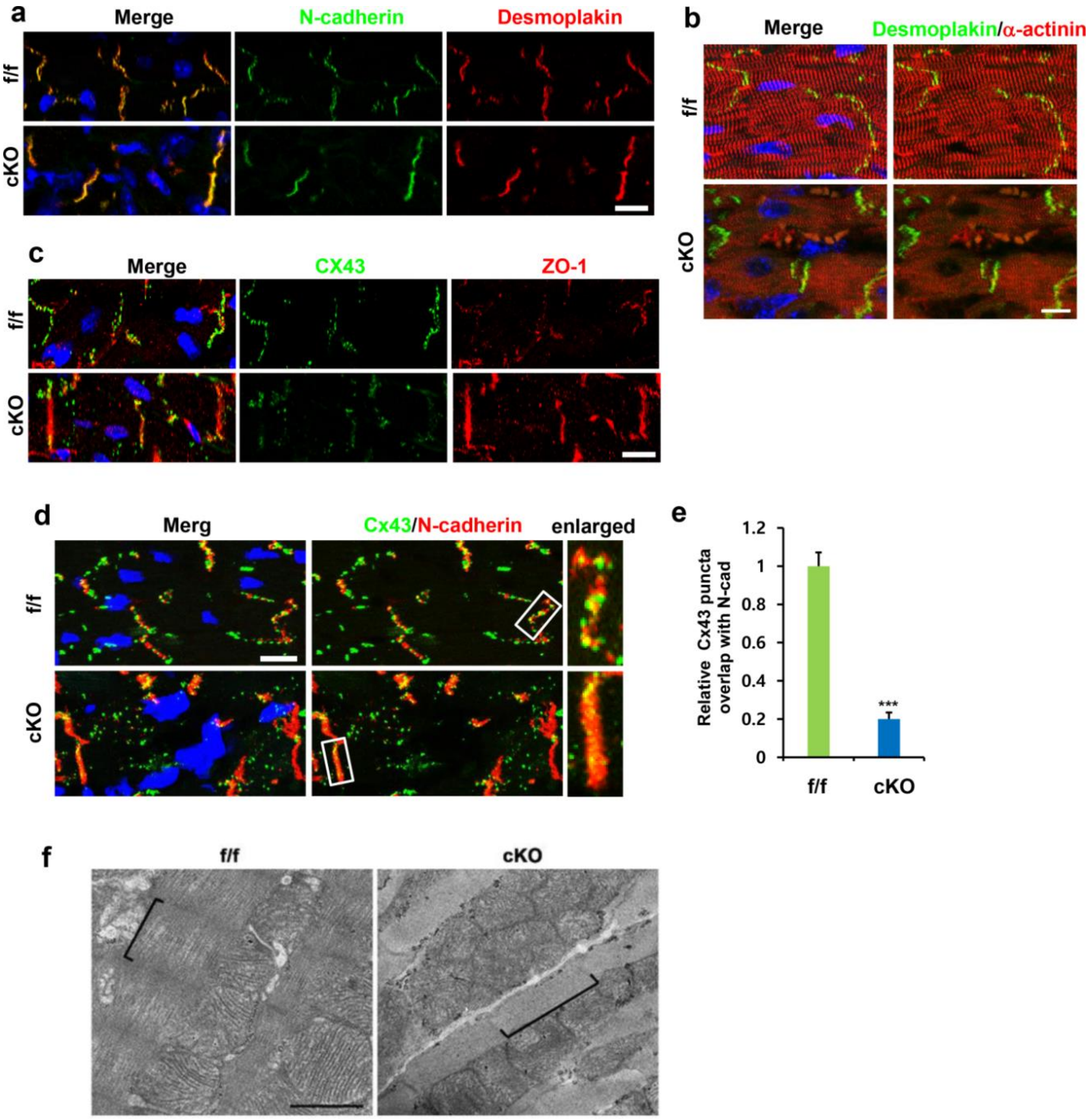

Supplementary figure 2

(**a**) Immunostaining of cardiac tissue of 8 weeks-old f/f and cKO mice for N-cadherin and Desmoplakin. Scale bar: 50µm. Note enhanced and broadened staining pattern of N-cadherin and Desmoplakin in cKO cardiomyocyte. (**b**) Immunohistochemistry for α-Actinin and Desmoplakin localized at intercalated disc in 8 weeks-old cardiac tissue Scale bar: 50 µm. Note the disarrayed α-actinin positive cytoskeletal structures. (**c**) Representative image for Cx43 and ZO-1 immunostaining. Scale bar: 50µm. Note thickened ZO-1 immunopositive signal at intercalated disc of cKO cardiomyocyte. (**d**) Immunostaining for Cx43 and N-cadherin as a marker of intercalated disc. Boxed areas are enlarged. Scale bar: 50µm. Note reduced Cx43 signal at intercalated disc and scattered Cx43 in the cytoplasmic area of cKO cardiomyocyte. (**e**) Quantification of Cx43 signal overlapping with N-cadherin signal. Mean values of at least 9 fields per each. n=3. Data represent means ±SD. ****P<0.001*. (**f**) Transmission electron microscopy picture showing alterations in cytoskeletal organization and mitochondria structures in 8 weeks-old cKO heart section, compared to the f/f. Scale bar: 1µm. The bracket depicts a sarcomere. Note the stretched sarcomere in cKO heart, relative to the f/f heart.

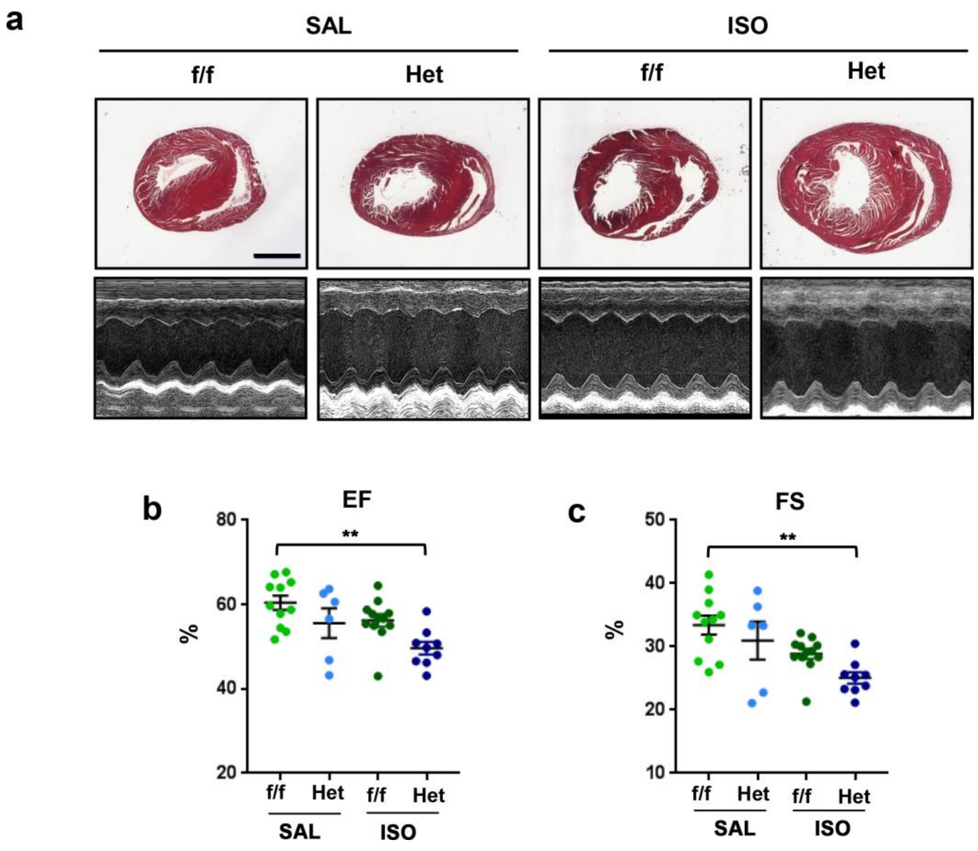

Supplementary figure 3

(**a**) The histological analysis and the short axis transthoracic M mode echocardiographic tracings from control f/f and PRMT1 heterozygous mice treated with isoproterenol for 2 weeks starting from 2 months of the age. Scale bar: 2mm. (**b, c**) The analysis of echocardiographic parameters; the ejection fraction and the fractional of shortening. n=11 (f/f, SAL), 6 (Het, SAL), 12 (f/f, ISO), 9 (Het, ISO). Data represent means ±SEM, one-way ANOVA. ***P<0.005*.

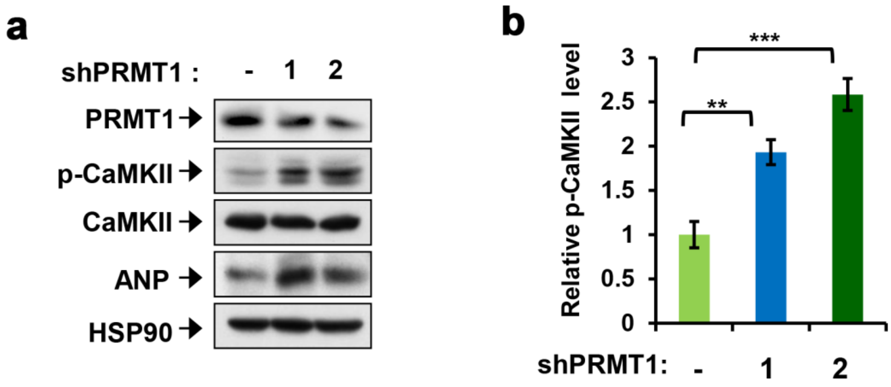

Supplementary figure 4

(**a, b**) Immunoblot analysis for PRMT1 and CaMKII in NRVM treated with PRMT1-targeted shRNAs. Relative levels of p-CaMKII. n=3. Data represent means ±SD. ***P<0.01*, ****P<0.001*, Student's *t*-test.

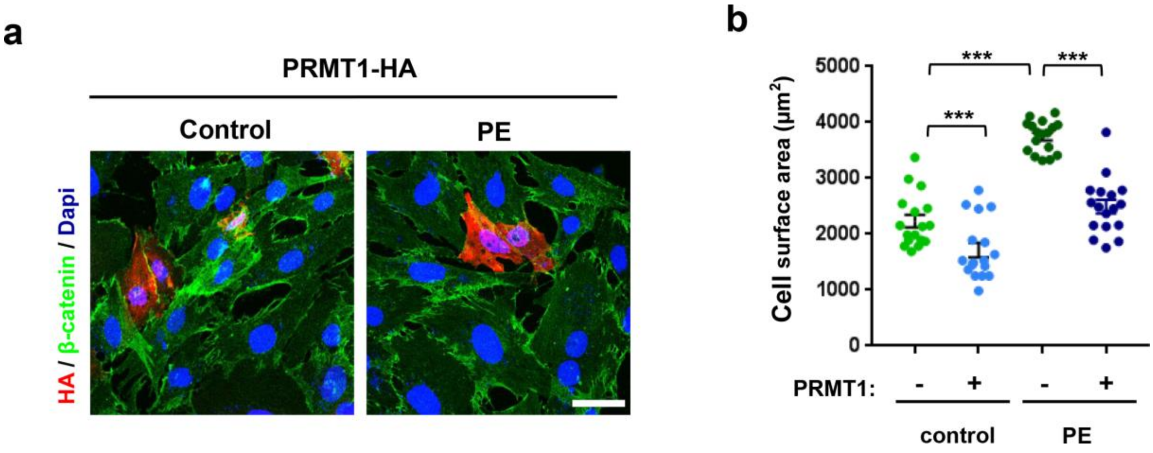

Supplementary figure 5

(**a**) Representative image of PRMT1-HA overexpressing H9C2 cardiomyocytes in response to vehicle or PE treatment. Scale bar: 50μm. (**b**) Quantification of surface area of cells similar shown in panel a. n=30. Data represent means ±SD. ****P<0.001*, Student's *t*-test.

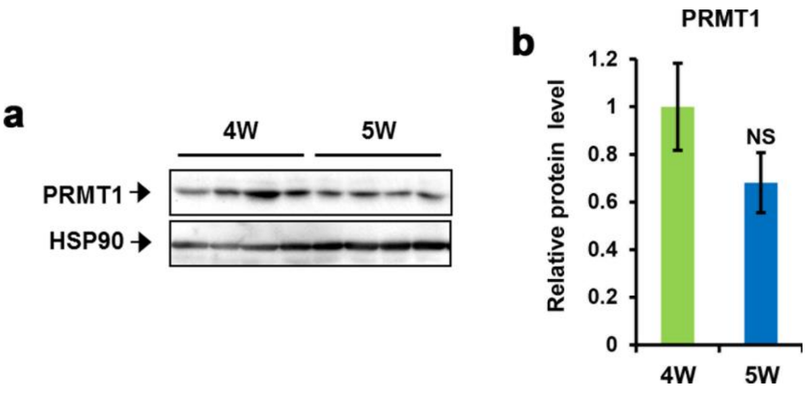

Supplementary figure 6

(a) Immunoblot analysis for PRMT1 proteins in heart lysates from 4 weeks and 5 weeks-old mice. (**b**) Quantification of the relative signal intensity shown in panel a. Data represent means ±SD. NS; not significant.

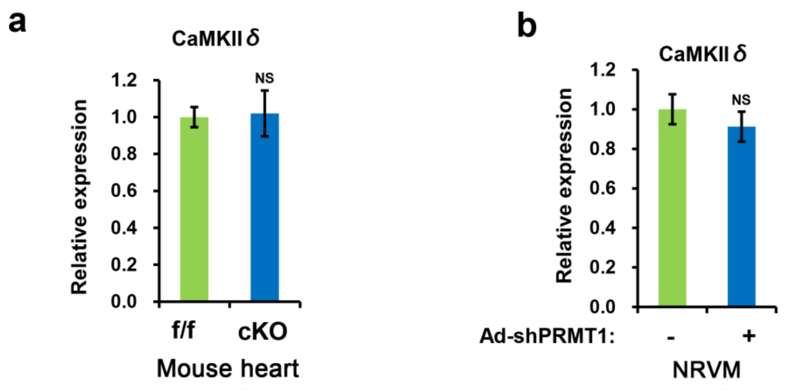

Supplementary figure 7

(**a, b**) The transcript level of CaMKIIδ was examined in f/f and cKO hearts (**a**) or control or shPRMT1-expressing NRVM cells (**b**) by quantitative RT-PCR. n=3. NS: not significant.

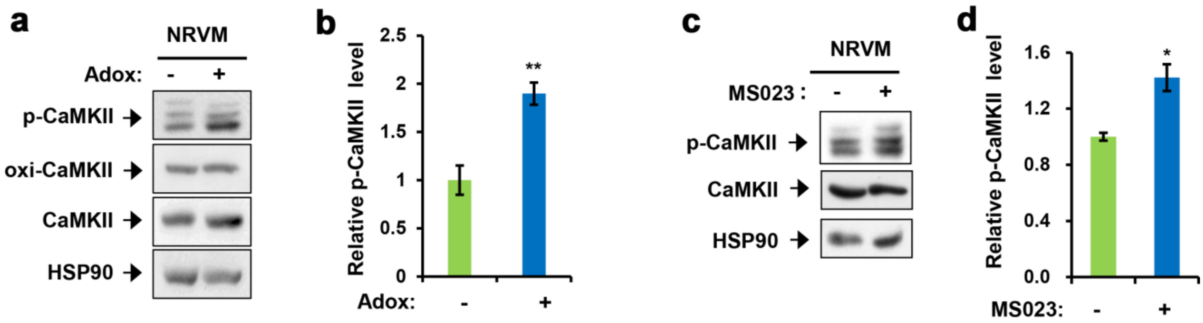

Supplementary figure 8

(**a, b**) Immunoblot analysis for CaMKII proteins in NRVM treated with a pan-PRMT inhibitor Adox for 4 hours and quantification of the relative signal intensity. The value from the vehicle treatment was set to 1. n=3. Data represent means ±SD. $^{**}P<0.01$, Student's *t*-test. (**c, d**) Immunoblot assay for CaMKII proteins in NRVM treated with MS023, an inhibitor for type1 PRMT family and quantification of the signal intensity of p-CaMKII in panel c. n=3. Data represent means ±SD. $^{*}P<0.05$, Student's *t*-test.

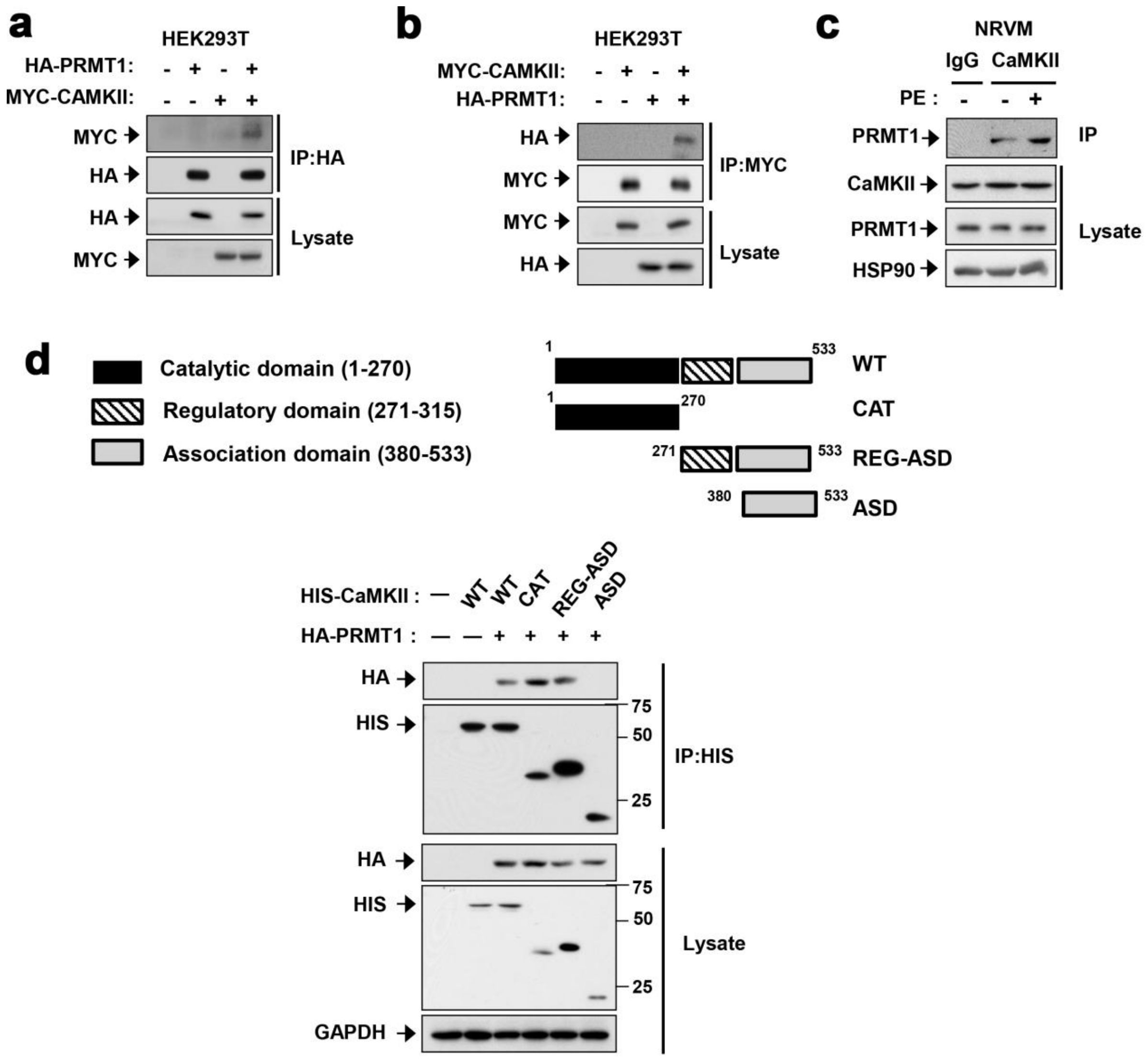

Supplementary figure 9

(**a, b**) Coimmunoprecipitation of PRMT1 and CaMKII in 293T cells transfected with HA-PRMT1 and Myc-CaMKII. (**c**) Coimmunoprecipitation of PRMT1 with CaMKII in NRVM in response to PE. (**d**) Coimmunoprecipitation of HIS-tagged CaMKII fragments (aa1-270, catalytic domain; 271-315, association domain, aa380-533; regulatory-association domain, aa-271-533) with HA-PRMT1.

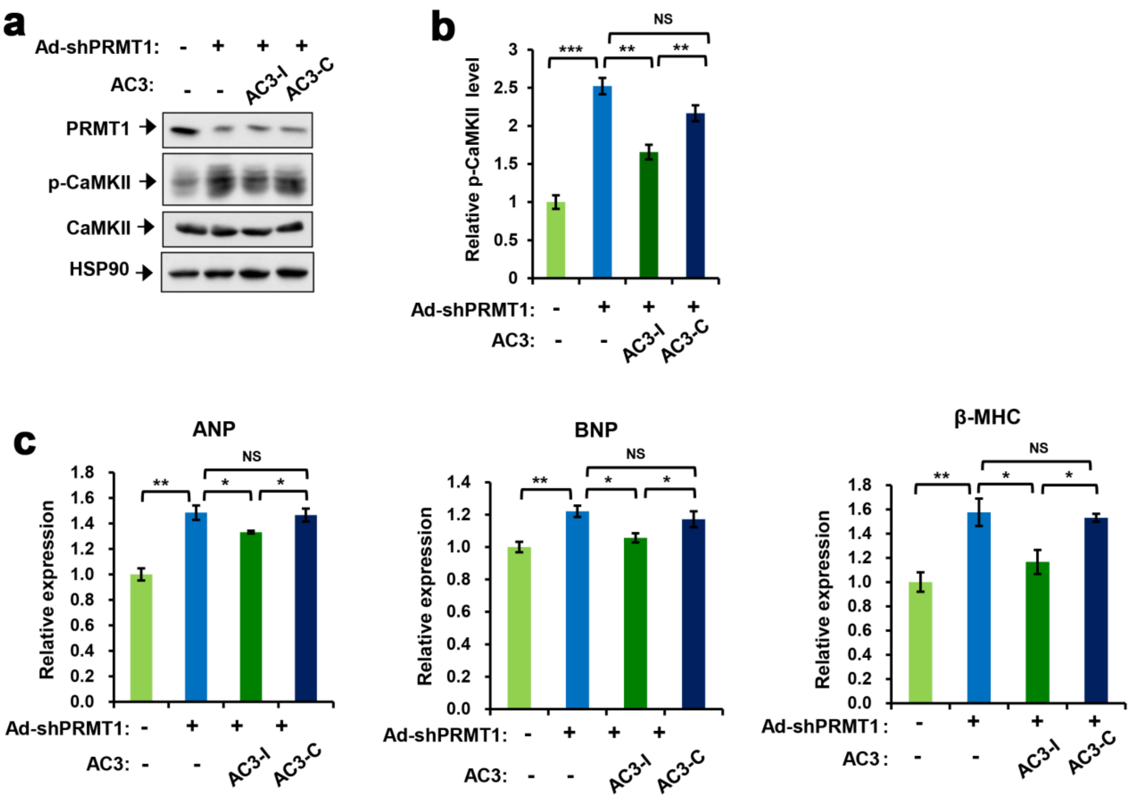

Supplementary figure 10

(**a**) Immunoblot analysis of Ad-control or Ad-shPRMT1-infected NRVM cells and treated with AC3-I and AC3-C peptides for 12 hours. (**b**) quantification of relative p-CaMKII level. n=3. Data represent means ±SD. $^{**}P<0.01$, $^{***}P<0.001$, Student's *t*-test. (**c**) qRT-PCR analysis of ANP, BNP and β-MHC levels in PRMT1-depleted NRVM treated with AC3 peptides. n=3. Data represent means ±SD. $^{*}P<0.05$, $^{**}P<0.01$.

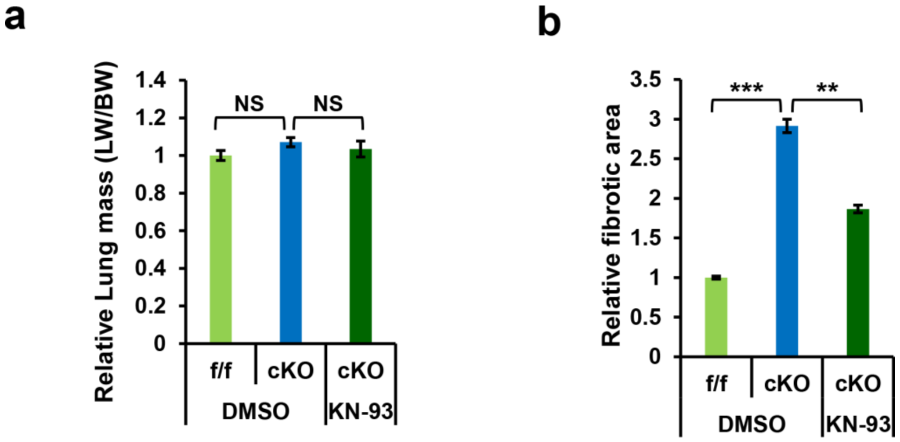

Supplementary figure 11

(**a**) Relative lung mass of f/f and cKO mice treated with vehicle or KN-93. Data represent means ±SD. NS: no significance. (**b**) Quantification of fibrotic area in panel i of figure 6. n=3. Data represent means ±SD. $^{**}P<0.01$, $^{***}P<0.001$, Student's *t*-test.

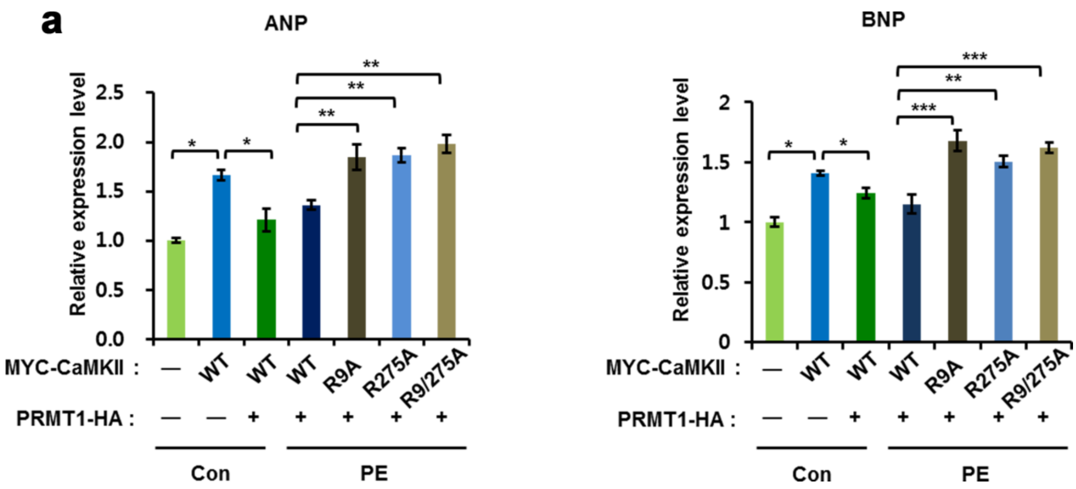

Supplementary figure 12

(**a**) Analysis of hypertrophic gene expression level by qRT PCR for WT and CaMKII mutant transfected NRVM in presence of PRMT1 overexpression and PE treatment for 12 hours. Data represent means ±SD. $^{*}P<0.05$, one-way ANOVA.

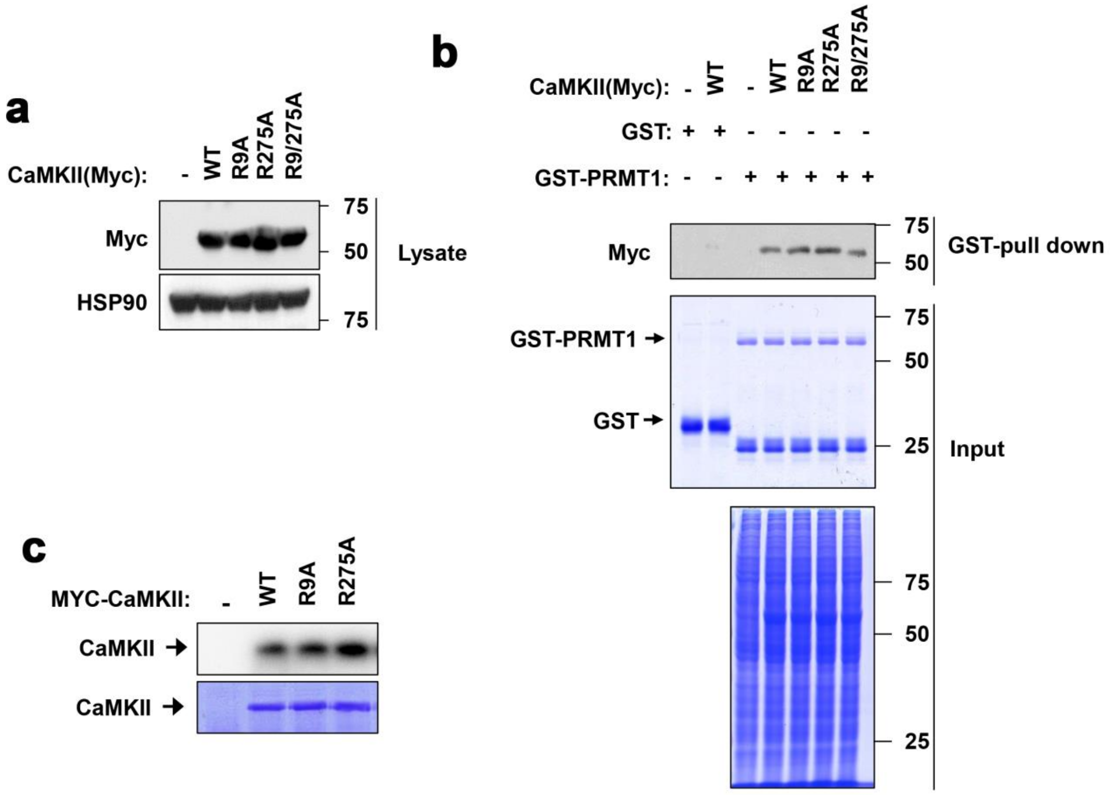

Supplementary figure 13**Supplementary figure 13**

(**a**) Immunoblot analysis for the expression of CaMKII proteins in 293T cells that were used for the pulldown assay. (**b**) Pull-down assay with purified GST and GST-tagged PRMT1 protein with 293T lysates shown in panel a. (**c**) In vitro kinase assay for autophosphorylation of CaMKII proteins.

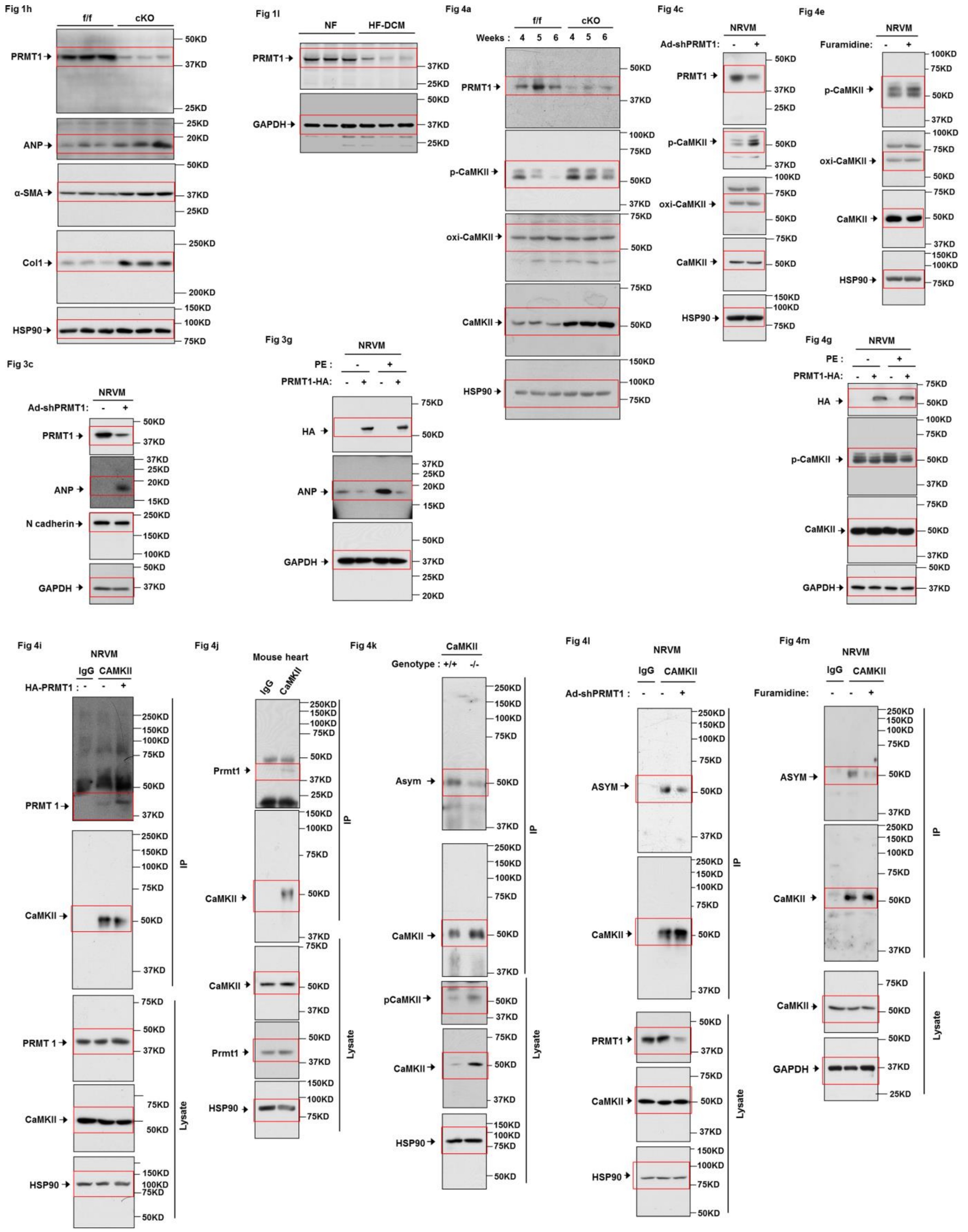

Supplementary figure 14

Uncropped scans of western blots from figure 1. h, l; figure 3. c, g; figure 4. a, c, e, g, i, j, k, l, m.

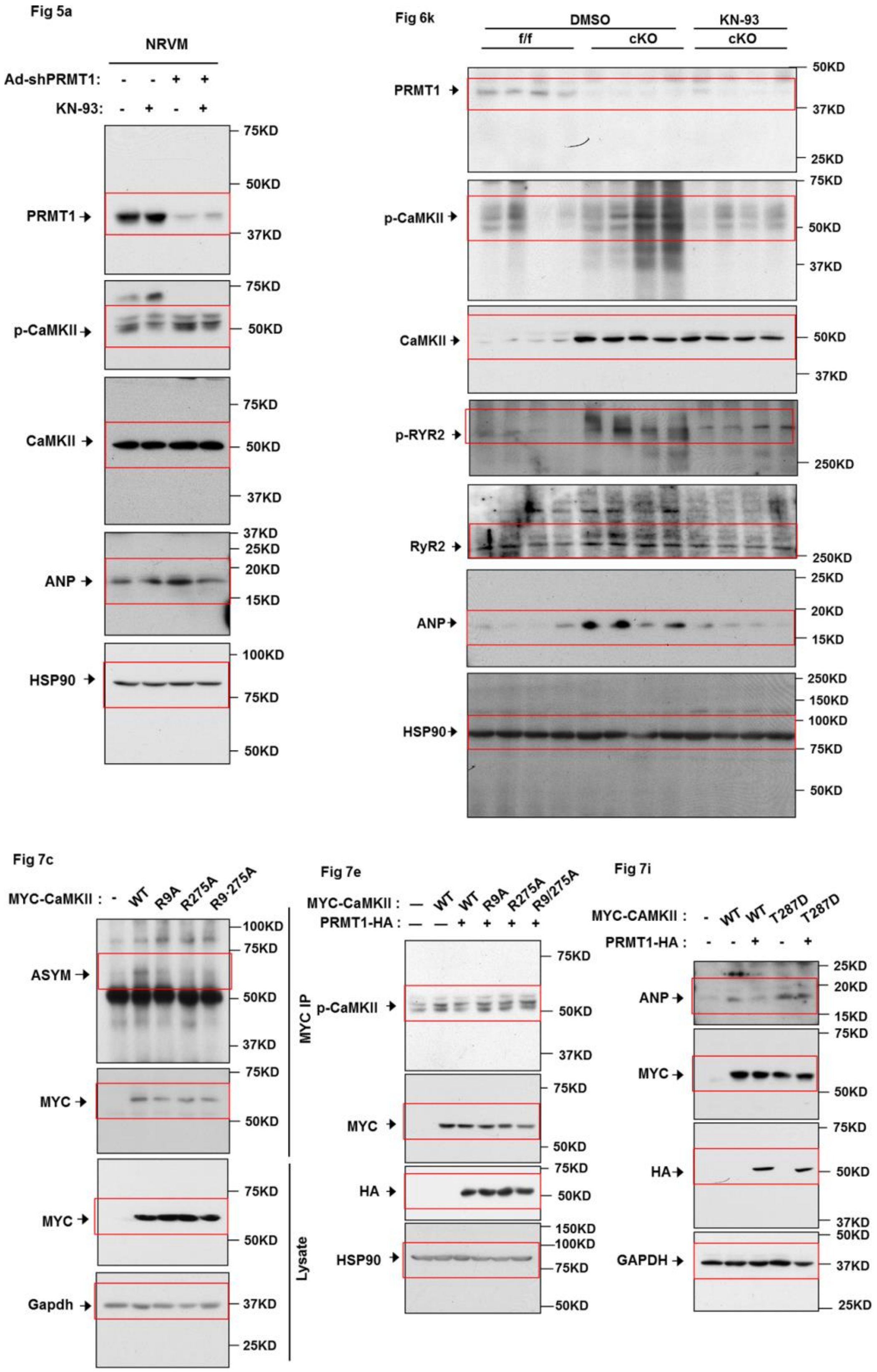

Supplementary figure 15

Uncropped scans of western blots from figure 5. a; figure 6. k; figure 7. c, e, i.

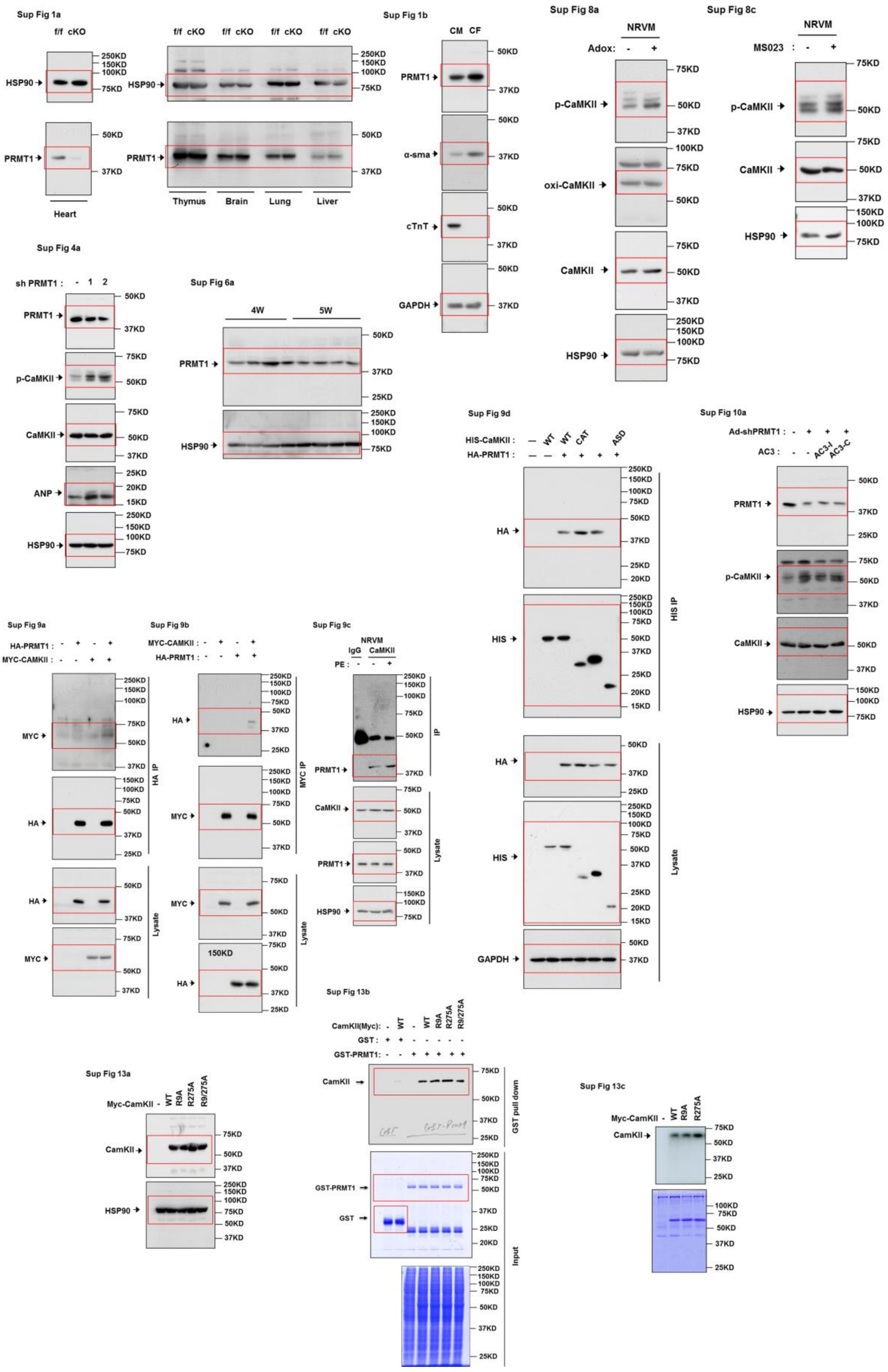

Supplementary figure 16

Uncropped scans of western blots from supplementary figure 1. a, b; supplementary figure 4. a; supplementary figure 6. a; supplementary figure 8. a, c; supplementary figure 9. a, b, c, d; supplementary figure 10. a; supplementary figure 13. a, b, c.

Supplementary Table 1. Primer sequences used in study.

Primer	Forward(5'→3')	Reverse(3'→5')
**PRMT1 flox**	GTG CTT GCC ATC AAG AGA TCC	ACA GCC GAG TAG CAA GGA GG
**Myosin heavy chain 6 (WT)**	CAA ATG TTG CTT GTC TGG TG	GTC AGT CGA GTG CAC AGT TT
**Myosin heavy chain 6 (TG)**	ATG ACA GAC AGA TCC CTC CTA TCT CC	CTC ATC ACT CGT TGC ATC ATC GAC
**ANP(rat)**	CCG TCT CAG AGA GAT GGA GG	ATC CTG TCA ATC CTA CCC CC
**BNP(rat)**	CAG CTC TCA AAG GAC CAA GG	GCA GCT TGA ACT ATG TGC CA
**β-MHC(rat)**	TGG CAC CGT GGA CTA CAA TA	TAC AGG TGC ATC AGC TCC AG
**CaMKII delta(rat)**	TGG CAT AGT TCA CAG GGA CC	TGC CAG CAA AAC CAA ACC AC
**GAPDH(rat)**	GAC ATG CCG CCT GGA GAA AC	AGC CCA GGAT GCC CTT TAG T
**ANP(mouse)**	CAG AAT CGA CTG CCT TTT CC	GGG GGT AGG ATT GAC AGG AT
**BNP(mouse)**	ACC CAG GCA GAG TCA GAA AC	ACA AGA TAG ACC GGA TCG GA
**CaMKII delta(mouse)**	AGG GAC CTG AAG CT GAG AA	GTG CCA GCA AAA CCA AAC CA
**GAPDH(mouse)**	CAG TGC CAG CCT CGT CCC GTA GA	CTG CAA ATG GCA GCC CTG GTG AC
**CaMKII delta R9A**	CTC GTC GGT GAA CGC GGT GCA GGT GGT G	CAC CAC CTG CAC CGC GTT CAC CGA CGA
**CaMKII delta R275A**	AGG CAA CAG TAG AAG CTT GAC AGA TCC ATG GGT GTT TCA GG	CCT GAA ACA CCC ATG GAT CTG TCA AGC TTC TAC TGT TGC CT
